# FUS ALS-causative mutations impair FUS autoregulation and splicing factor networks through intron retention

**DOI:** 10.1093/nar/gkaa410

**Published:** 2020-06-01

**Authors:** Jack Humphrey, Nicol Birsa, Carmelo Milioto, Martha McLaughlin, Agnieszka M Ule, David Robaldo, Andrea B Eberle, Rahel Kräuchi, Matthew Bentham, Anna-Leigh Brown, Seth Jarvis, Cristian Bodo, Maria G Garone, Anny Devoy, Gianni Soraru, Alessandro Rosa, Irene Bozzoni, Elizabeth M C Fisher, Oliver Mühlemann, Giampietro Schiavo, Marc-David Ruepp, Adrian M Isaacs, Vincent Plagnol, Pietro Fratta

**Affiliations:** Department of Neuromuscular Diseases, UCL Queen Square Institute of Neurology, University College London, London WC1N 3BG, UK; UK Dementia Research Institute; Department of Neurodegenerative Diseases, UCL Queen Square Institute of Neurology, University College London, London WC1N 3BG, UK; UCL Genetics Institute, University College London, London WC1E 6BT, UK; Department of Neuromuscular Diseases, UCL Queen Square Institute of Neurology, University College London, London WC1N 3BG, UK; UK Dementia Research Institute; UK Dementia Research Institute; Department of Neurodegenerative Diseases, UCL Queen Square Institute of Neurology, University College London, London WC1N 3BG, UK; Department of Neuromuscular Diseases, UCL Queen Square Institute of Neurology, University College London, London WC1N 3BG, UK; Department of Neuromuscular Diseases, UCL Queen Square Institute of Neurology, University College London, London WC1N 3BG, UK; UK Dementia Research Institute; Department of Neurodegenerative Diseases, UCL Queen Square Institute of Neurology, University College London, London WC1N 3BG, UK; Department of Chemistry and Biochemistry, University of Bern, Freiestrasse 3, Bern 3012, Switzerland; Department of Chemistry and Biochemistry, University of Bern, Freiestrasse 3, Bern 3012, Switzerland; Department of Neuromuscular Diseases, UCL Queen Square Institute of Neurology, University College London, London WC1N 3BG, UK; Department of Neuromuscular Diseases, UCL Queen Square Institute of Neurology, University College London, London WC1N 3BG, UK; UCL Genetics Institute, University College London, London WC1E 6BT, UK; Department of Neuromuscular Diseases, UCL Queen Square Institute of Neurology, University College London, London WC1N 3BG, UK; UK Dementia Research Institute; UCL Genetics Institute, University College London, London WC1E 6BT, UK; Department of Neuromuscular Diseases, UCL Queen Square Institute of Neurology, University College London, London WC1N 3BG, UK; Sapienza University of Rome, Rome 00185, Italy; Department of Neuromuscular Diseases, UCL Queen Square Institute of Neurology, University College London, London WC1N 3BG, UK; UK Dementia Research Institute; Maurice Wohl Clinical Neuroscience Institute, King’s College London, London SE5 9RT, UK; Department of Neurosciences, Università degli Studi di Padova, Padova 35121, Italy; Sapienza University of Rome, Rome 00185, Italy; Center for Life Nano Science, Istituto Italiano di Tecnologia, Rome 00161, Italy; Sapienza University of Rome, Rome 00185, Italy; Center for Life Nano Science, Istituto Italiano di Tecnologia, Rome 00161, Italy; Department of Neuromuscular Diseases, UCL Queen Square Institute of Neurology, University College London, London WC1N 3BG, UK; Department of Chemistry and Biochemistry, University of Bern, Freiestrasse 3, Bern 3012, Switzerland; Department of Neuromuscular Diseases, UCL Queen Square Institute of Neurology, University College London, London WC1N 3BG, UK; UK Dementia Research Institute; Discoveries Centre for Regenerative and Precision Medicine, University College London Campus, London WC1N 3BG, UK; UK Dementia Research Institute; Maurice Wohl Clinical Neuroscience Institute, King’s College London, London SE5 9RT, UK; UK Dementia Research Institute; Department of Neurodegenerative Diseases, UCL Queen Square Institute of Neurology, University College London, London WC1N 3BG, UK; UCL Genetics Institute, University College London, London WC1E 6BT, UK; Department of Neuromuscular Diseases, UCL Queen Square Institute of Neurology, University College London, London WC1N 3BG, UK

## Abstract

Mutations in the RNA-binding protein FUS cause amyotrophic lateral sclerosis (ALS), a devastating neurodegenerative disease. FUS plays a role in numerous aspects of RNA metabolism, including mRNA splicing. However, the impact of ALS-causative mutations on splicing has not been fully characterized, as most disease models have been based on overexpressing mutant FUS, which will alter RNA processing due to FUS autoregulation. We and others have recently created knockin models that overcome the overexpression problem, and have generated high depth RNA-sequencing on FUS mutants in parallel to FUS knockout, allowing us to compare mutation-induced changes to genuine loss of function. We find that FUS-ALS mutations induce a widespread loss of function on expression and splicing. Specifically, we find that mutant FUS directly alters intron retention levels in RNA-binding proteins. Moreover, we identify an intron retention event in FUS itself that is associated with its autoregulation. Altered FUS levels have been linked to disease, and we show here that this novel autoregulation mechanism is altered by FUS mutations. Crucially, we also observe this phenomenon in other genetic forms of ALS, including those caused by TDP-43, VCP and SOD1 mutations, supporting the concept that multiple ALS genes interact in a regulatory network.

## INTRODUCTION

Amyotrophic lateral sclerosis (ALS) is a relentlessly progressive neurodegenerative disorder characterized by loss of motor neurons, leading to muscle paralysis and death (1). About 5–10% of cases are inherited in an autosomal dominant fashion (1). Numerous genes have been identified as disease-causative, and have been central to the understanding of pathogenesis. RNA-binding proteins (RBPs), most prominently TDP-43 and FUS, have been identified as a major category of causative genes in familial ALS (2,3). Both TDP-43 and FUS have multiple roles in RNA metabolism, including transcription, splicing, polyadenylation, miRNA processing and RNA transport (4–9). Although both proteins are predominantly localized to the nucleus, in post-mortem brain tissue from mutation carriers a mislocalization of the affected protein to the cytoplasm can be observed. This shared pathology has suggested both a nuclear loss of function and a cytoplasmic gain of toxic function to play a role in familial FUS and TDP-43 ALS (9). Although many studies have investigated the physiological functions of FUS and TDP-43 through knockout and overexpression experiments (10–23), the effect of disease-causing mutations on RNA splicing has been more challenging to investigate. Due to the fact that both proteins are very sensitive to dosage changes and very tightly regulated (24,25), mutation overexpression models are unfit to address these questions.

**Figure 1.**
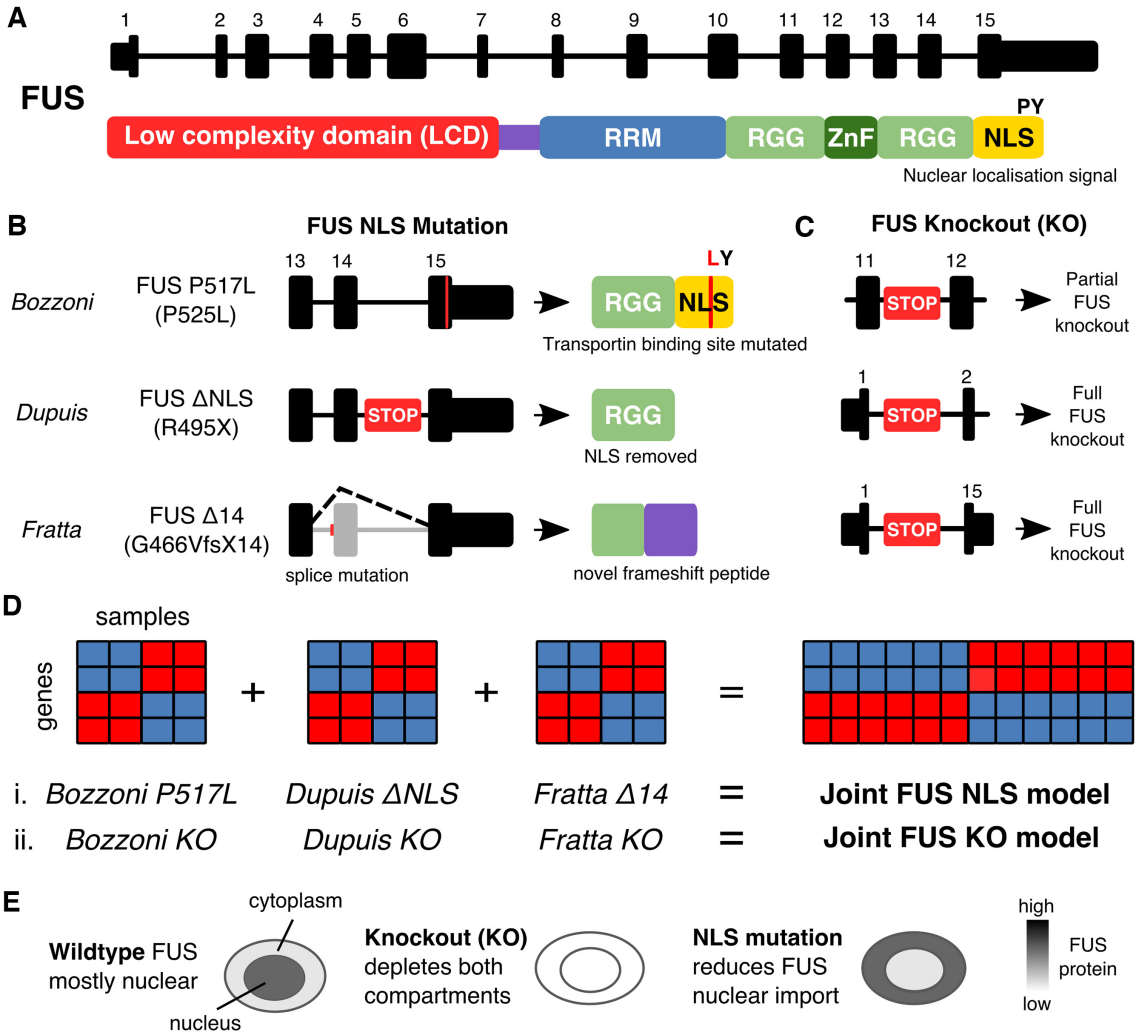
Illustration of the models and mutations used in this study. (**A**) Transcript and protein structure of FUS. The major transcript encoding the FUS protein in humans and mice is comprised of 15 exons. FUS protein contains a low complexity domain (LCD), an RNA recognition motif (RRM) domain, two Arginine-Glycine-Glycine (RGG) domains, a zinc finger domain (Znf) and a nuclear localization signal (NLS) (29). (**B**) The three mouse FUS NLS mutations used in this study. The Bozzoni group knocked in a point mutation to create the FUS P517L line, a missense mutation equivalent to the human ALS P525L mutation. The Dupuis group created a FUS ΔNLS line where the entire NLS is removed. We have used the FUS-Δ14 mouse, where a frameshift mutation leads to the skipping of exon 14 and a frameshifting of the remaining NLS sequence. (**C**) The FUS knockout alleles used by each group. STOP here refers to the GeneTrap transgene used. (**D**) Schematic explaining the two joint models created from the six individual gene expression datasets. (**E**) Proposed rationale for study. In wild-type cells FUS protein is predominantly nuclear but can shuttle to and from the cytoplasm. When FUS is knocked out it will be reduced in both compartments but if the NLS is mutated or deleted then FUS will accumulate in the cytoplasm due to reduced nuclear import.

We and others have used mice carrying mutations in the endogenous *Tardbp* gene to show that TDP-43 mutations induce a splicing gain of function (26,27). Here, we use our novel knockin mouse model of FUS-ALS, FUS-Δ14 (28), in combination with data from other physiological mouse and cellular models of FUS-ALS, to address the impact of ALS-causing FUS mutations on RNA metabolism, and splicing in particular. Although mutations have been observed throughout the FUS gene, the most aggressive FUS ALS-causing mutations cluster in the C-terminal region of the protein, where the nuclear localization signal (NLS) resides (Figure 1A) (29). These mutations affect the binding of the nuclear localization signal by the nuclear import receptor Transportin (TNPO1) and induce an increase in cytoplasmic localization of the protein (29,30).

We find that FUS NLS mutations induce a splicing loss of function, particularly through intron retention events. These events are enriched in transcripts encoding RBPs, including FUS itself. We propose intron retention to be a mechanism whereby FUS autoregulates its own expression levels. Finally, we show that this regulatory splicing event is altered in different ALS model systems. These findings shed light on primary changes caused by ALS mutations, on how RBPs act as a network to regulate each other, and on the diverse mechanisms of RBP autoregulation. This is of central importance for understanding the vicious cycle of cytoplasmic accumulation of RBPs driving further RBP overexpression and mislocalization that ensues in disease (31) and is a target for therapeutic strategies.

## MATERIALS AND METHODS

### External datasets

FUS-ΔNLS and FUS KO mouse brain samples with their respective controls (32) were downloaded from SRA accession SRP070906. FUS P517L mutants, FUS KO and shared control samples from mouse motor neurons (33) were downloaded from SRA accession SRP111475. Data from a series of motor neuron differentiation experiments, where induced pluripotent stem cells with and without VCP mutations were differentiated to mature motor neurons with RNA-seq libraries created from cells taken at 0, 7, 14, 21 and 35 days following differentiation (34), were downloaded from the Gene Expression Omnibus with accession GSE98290. Stem cell-derived human motor neurons carrying SOD1 A4V mutations and their isogenic controls (35) were downloaded from GSE54409. FUS, U2AF65 and TDP-43 mouse iCLIP (6) clusters were downloaded from http://icount.biolab.si/. TDP-43, FUS, EWSR1 and TAF15 CLIP data from human and mouse were downloaded from POSTAR (36). When the same CLIP sample had been processed with more than one peak caller, the Piranha caller was selected for presentation. Post-mortem human brain RNA-seq from Frontotemporal Dementia with TDP-43 or Tau pathology (37) was downloaded from GSE90696. Post-mortem human brain RNA-seq from sporadic and C9orf72 ALS patients (38) was downloaded from GSE67196.

### Mouse lines

FUS-Δ14 mice (B6N;B6J-Fus^tm1Emcf/H^) were previously described (28). FUS knockout mice were obtained from the Mouse Knockout Project (Fustm1(KOMP)Vlcg). All mouse lines were backcrossed onto C57BL/6J animals for more than five generations. All procedures for the care and treatment of animals were in accordance with the Animals (Scientific Procedures) Act 1986 Amendment Regulations 2012.

### RNA sequencing

For RNA sequencing experiments FUS-Δ14 or KO heterozygous and homozygous mice were compared to their respective wild-type littermates. Spinal cords were collected from E17.5 mouse embryos. Tissues were snap frozen, genotyped and total RNA was extracted from the appropriate samples using Qiazol followed by the mini RNAeasy kit (Qiagen). RNA samples used for sequencing all had RIN values of 9.9 or 10. cDNA libraries were made at the Oxford Genomics facility using a TruSeq stranded total RNA RiboZero protocol (Illumina). Libraries were sequenced on an Illumina HiSeq to generate paired-end 150 bp reads.

### RNA-seq data processing

Mouse data were aligned to the mm10 build of the mouse genome and human data aligned to the hg38 build of the human genome using STAR (v2.4.2). Prior to differential expression analysis, reads were counted across genes with HTSeq. All code for exploratory data analysis, statistical testing and visualization were carried out in the R statistical programming language, using the tidyverse suite of packages. Data visualization and figure creation were aided by the patchwork, stargazer, ggbeeswarm and ggrepel packages. All R code written for the project is publicly available as interactive Rmarkdown notebooks (https://github.com/jackhump/FUS_intron_retention).

### Joint modeling of differential expression

Each dataset consists of FUS knockout samples, FUS NLS mutation samples and wild-type controls. In the Bozzoni dataset, the controls are shared but in the other two datasets the knockout and mutation samples have their own separate controls for use in two-way comparisons. Differential gene expression was tested with DESeq2. Initially each comparison (wild-type versus knockout or wild-type versus mutation) was performed separately for each dataset, creating six individual analyses. To boost power and create a set of high confidence changes, two joint models were created using either the knockout (KO) or mutation (MUT) samples with their specific controls. The joint model uses all the samples of the same comparison together in a general linear model with a dataset-specific covariate. DESeq2 uses a Bayesian shrinkage strategy when estimating the log_2_ fold change. For each gene the estimated log_2_ fold change is a combination of the three individual datasets. Genes are reported as significantly differentially expressed at a false discovery rate (FDR) threshold of 0.05 (39). For plots, gene expression values are raw counts multiplied by each sample’s size factor generated by DESeq2. These normalized counts are then normalized to the wild-type samples for each dataset to visualize the relative change in expression.

To assess the level of overlap between the FUS KO and FUS NLS joint models, two different overlap thresholds were employed. The first, a more conservative threshold, depends on a gene being significant at FDR < 0.05 in both datasets. The second, more relaxed threshold, calls a gene as significant if it falls below FDR < 0.05 in one dataset and has an uncorrected *P*-value < 0.05 in the other.

### Joint modeling of splicing

SGSeq splicing analysis was performed on all the samples together to discover and classify all potential splicing events using the default parameters for finding both annotated and novel splicing. Differential splicing for individual comparisons and joint models with a dataset-specific covariate were performed using DEXSeq. The same overlap threshold strategies were employed as for differential gene expression. SGSeq looks for all potential splicing events in each sample and then counts the reads supporting either the inclusion or exclusion of that splicing variant. Percentage Spliced In (PSI) values (40) for each splicing variant in each sample were calculated by taking the read counts supporting the inclusion event and dividing by the total reads in that event.

### Analysis of FUS splicing in other datasets

Details of other datasets used are contained in Supplementary Table S8. All datasets were processed using the same pipeline as the FUS mouse data. SGSeq was used to quantify and test FUS splicing.

### Functional analysis of genes and splicing events

iCLIP data on FUS and U2AF65 from mouse brain (6) were reprocessed by the iCOUNT iCLIP analysis pipeline (http://icount.biolab.si/), and the set of FUS iCLIP clusters that passed enrichment against background at FDR < 0.05 were downloaded. Only iCLIP clusters with a minimum of two supporting reads were kept. Untranslated region (UTR) and coding exon (CDS) annotation were taken from GENCODE mouse (comprehensive; mouse v12). Any intron retention, nonsense mediated decay or ‘cds end nf’ transcripts were removed. UTR coordinates were split into 5′ and 3′ UTR based on whether they overlapped an annotated polyadenylation site or signal (GENCODE mouse v18 polyadenylation annotation). 3′ UTRs were extended by 5 kilobases downstream to capture any unannotated sequence. Introns were defined as any gaps in the transcript model between CDS and UTR coordinates. Promoter-antisense coordinates were taken by flanking the 5′ UTR sequence by 5 kb upstream and inverting the strand. Overlaps between iCLIP clusters and genomic features were created for each set of differentially expressed genes, split into upregulated (log_2_ fold change > 0) or downregulated (log_2_ fold change < 0). Overlaps were done in a strand-specific manner, with only iCLIP clusters in the same direction being used.

Whether an iCLIP cluster overlaps a genomic region depends on both the affinity of the chosen protein for RNA sequence of the motif and the abundance of the RNA in the cell (41). In addition, a longer region would be more likely to overlap an iCLIP cluster by random chance than a shorter region. When comparing sets of genomic regions, whether genes or splicing events, this must be taken into account.

To test for enrichment of FUS iCLIP clusters in upregulated and downregulated genes, each set of tested genes was compared to a set of null genes with no evidence of differential expression (*P* > 0.05 in both models). The null set was then restricted to genes with both length and expression values that were within the first and third quartile of those of the test gene set. The expression values were calculated by taking the mean number of reads covering each gene in the Fratta wild-type samples, with each sample read count first normalized by the library size factor for each sample calculated by DESeq2. The proportion of each set of genes overlapping an iCLIP peak was then compared to that of the null set with a χ^2^ test of equal proportions.

For the splicing events found in the joint models, enrichment tests were performed for different genomic features. For these tests, the coordinates of the entire encompassing intron were used for each splicing variant. Each test set of splicing events was compared to a matched set of null splicing events where *P* > 0.05 in both joint models. The null events were chosen to have length and expression levels within the first and third quartiles of that of the test set. Proportions of overlap with iCLIP clusters between splicing events and the null set were tested using a χ^2^ test of equal proportions. As a positive control in both analyses, the same overlaps were computed with iCLIP clusters from U2AF65, also from (6).

Per nucleotide phyloP conservation scores (42) comparing mouse (mm10) with 60 other vertebrates were downloaded from UCSC. The median phyloP score was calculated for each splicing variant and compared.

### RT-PCR–intron retention validation

Primers were designed using Primer3 and *in silico* PCR (UCSC). For both human and mouse FUS, the forward primer was designed for exon 6 and the reverse primer designed to span the spliced exon 8/9 junction to preferentially amplify spliced FUS mRNA. An additional third primer was designed to amplify a section of either intron 6 or intron 7. Primer sequences are listed in Supplementary Tables S5 and S6.

Cells were obtained from mouse spinal cord and/or cultured mouse embryonic fibroblasts resuspended in Trizol (Thermo Fisher Scientific). RNA was extracted using miRNeasy Mini Kit (Qiagen) following the manufacturer’s instructions. cDNA was obtained from extracted RNA using SuperScript IV Reverse Transcriptase kit (Thermo Fisher). Briefly, a mix was made of RNA template (500 ng for mouse brain; 100 ng for cultured cells (cycloheximide treatment)), 10 mM dNTP, 50 mM oligo d(T)_20_, 50 mM random hexamer followed by 5 min of incubation at 65°C and 1 min in ice. Mix was then complemented with 5X SuperScript IV Reverse Transcriptase buffer, 100 nM DTT, RNase OUT and SuperScript IV Reverse Transcriptase buffer followed by incubation at 23, 55 and 80°C, 10 min each.

RT-PCR was carried out using 10X AccuPrime Taq DNA polymerase mastermix system (Invitrogen). Each PCR reaction mix contained 5 ng of gDNA, 10 mM of forward and reverse primers. cDNA was amplified with the following conditions: Intron 6 retention: One cycle of 5 min at 95°C, followed by 30 cycles of 30 s at 95°C, 30 s at 56°C and 30 s at 68°C, and finishing with 5 min incubation at 68°C. Intron 7 retention: One cycle of 5 min at 95°C, followed by 30 cycles of 30 s at 95°C, 30 s at 61°C and 30 s at 68°C, and finishing with 5 min incubation at 68°C. Srsf7 NMD positive control: One cycle of 5 min at 95°C, followed by 35 cycles of 30 s at 95°C, 30 s at 58°C and 15 s at 68°C, and finishing with 5 min incubation at 68°C. Amplified products were finally visualized using Agilent 4200 TapeStation System following the manufacturer’s instructions. Results were analyzed on TapeStation analysis software (Agilent). Intron retention events are plotted as the percentage of the total integrated area corresponding to the intron retention product. One- or two-way ANOVA designs were employed with pairwise *t*-tests with Holm correction for multiple testing. For the RT-PCR on human fibroblasts, four technical replicates were obtained from two independent cell culture experiments, performed at different time points and derived from the same original two human samples (referred as +/+ and G496Gfs/+).

### Nuclear–cytoplasmic fractionation

Nuclear and cytoplasmic fractions were obtained from DIV7 murine primary ventral horn cultures cultured from WT E12-14 embryos. Briefly, the cells were washed with PBS then treated with Accutase (Innovative Cell Technologies) and TrypLE Express (Thermo Fisher) consecutively for 5 min each before lysis in Nuclei EZ lysis buffer (Sigma) for 5 min on ice. The lysates were then centrifuged at 500 *g* for 5 min and the supernatant was extracted and saved as the cytoplasmic fraction. The nuclear pellet was resuspended by gently triturating 5–8 times in freshly prepared Nuclei EZ lysis buffer before centrifugation again at 500 *g* for 5 min. The supernatant was discarded, and the nuclear pellet was resuspended in RIPA buffer for 5 min on ice. Nuclear DNA was sheared by passing the nuclear lysate through a 25 gauge needle 8–10 times. The nuclear and cytoplasmic lysates were then centrifuged again at 13 000 *g* for 5 min. The supernatants were added to an appropriate volume of TRIzol LS (Thermo Fisher) for RNA extraction. Equal amounts of RNA were reverse transcribed using IV VILO kit (Thermo Fisher) according to the manufacturer’s protocol. cDNA was used for end point PCR using Go-Taq Polymerase (Promega). Bands were resolved on an agarose gel and imaged using GelDoc (Bio-Rad). The same 3-primer design was used to amplify Fus with intron 6 or 7 retention. As a positive control, the nuclear RNA *Xist* was amplified with primers from (43).

*Xist* nuclear RNA PCR: One cycle of 5 min at 95°C, followed by 30 cycles of 30 s at 95°C, 30 s at 60°C and 30 s at 72°C, and finishing with 5 min incubation at 72°C. FUS Introns 6 and 7 retention PCR: One cycle of 5 min at 95°C, followed by 30 cycles of 30 s at 95°C, 30 s at 58°C and 20 s at 72°C, and finishing with 5 min incubation at 72°C.

### Cycloheximide treatment

Mouse embryonic fibroblasts were treated with 100 μg/ml cycloheximide (Sigma) for 6 h before RNA was extracted with Trizol (Thermo Fisher) and RT-PCR performed as before. As a positive control, primers targeting the NMD-sensitive exon 4 of *Srsf7* were used from (44).

### Plasmids

pLVX-EF1a-TS-EGFP-IRES-Puro was cloned by introducing an N-terminal Twin-Strep (TS)-tagged EGFP cDNA (DNA String byGeneArt, Life Technologies) into the EcoRI and BamHI sites of pLVX-EF1a-IRES-Puro (Clontech, Cat. Nr. 631988). pLVX-EF1a-TS-OPT-FUS-IRES-Puro was cloned by introducing an N-terminal Twin-Strep (TS)-tagged codon optimized FUS cDNA (Gene synthesis by GeneArt, Life Technologies) into the EcoRI and BamHI sites of pLVX-EF1a-IRES-Puro (Clontech, Cat. Nr. 631988).

### Stable cell line generation

293T cells were cultured in DMEM/F12 supplemented with 10% heat-inactivated, tetracycline-free fetal calf serum (FCS) (Contech, Cat. Nr. 631105), penicillin (100 IU/ml)/streptomycin (100 μg/ml) (Amimed, Bioconcept Cat. Nr. 4-01F00-H). One day prior to transfection, approximately 5 × 10^6^ HEK293T cells were plated in 150 cm^2^ flasks. About 28 mg of the pLVX-EF1a vectors and 144 ml of the fourth generation Lenti-X HTX Packaging Mix (Clontech, Cat. Nr. 631249) were transfected using the Xfect transfection reagent (Clontech, Cat. Nr. 631317). Twenty-four hours post transfection the medium was exchanged. About 48, 72 and 96 hours post transfection viral particle containing supernatants were harvested and filtered through a 0.45 μm PES syringe filter (Membrane Solutions, Cat. Nr. SFPES030045S) followed by a 6-fold concentration using Lenti-X-Concentrator (Clontech, Cat. Nr. 631232) according to the manufacturer’s instructions.

One day before transduction, 2 × 10^5^ HeLa cells were seeded into four wells of a six-well plate. The next day, the cells were exposed to 1 ml concentrated viral supernatant in a total volume of 2 ml DMEM+/+ supplemented with 10 μg/ml Polybrene (Sigma Aldrich, Cat. Nr. 107689) to increase lentiviral transduction efficiency. The following 2 days, the same procedure was carried out with virus from the second and third harvest, respectively. Finally, the transduced cells were expanded under constant puromycin selection at 2 μg/ml.

### Knockdown of UPF1 by siRNAs

Knockdown of UPF1 was carried out in three different HeLa cell lines: wild-type cells, cells containing stably integrated GFP reporter gene, and cells containing stably integrated codon-optimized FUS reporter gene. Knockdown was achieved using siRNAs for UPF1 (GAUGCAGUUCCGCUCCAUUdTdT) and scrambled control sequence (AGGUAGUGUAAUCGCCUUGdTdT, Microsynth, CH). In short, 2–3 × 10^5^ cells were seeded into the well of a six-well plate and transfected the following day with 40 nM siRNAs using Lullaby (OZ Biosciences) according to the manufacturer’s protocol. After 48 h, cells were re-transfected with 40 nM siRNAs and harvested 48 h after the second siRNA transfection. Until harvest, cells were split to avoid overgrowth of the cell culture. The efficiency of the knockdown was assessed by western blotting.

### RT-qPCR

RNA analysis was performed according to (45). Briefly, harvested cells were lysed in Trizol reagent (Thermo Fisher) and RNA was isolated according to standard protocol. Prior to reverse transcription (RT), DNase treatment was performed using Turbo DNA-free kit (Invitrogen) to avoid any DNA contamination. cDNA was synthesized using AffinityScript Multiple Temperature Reverse Transcriptase (Agilent) and RT control samples (without addition of RT) were included for each sample. The cDNA was measured in triplicates by RT-qPCR (reaction volume 15 μl) using Rotor-Gene Q (Qiagen) and Brilliant III Ultra-Fast SYBR Green qPCR Master Mix (Agilent). Oligonucleotides (final concentration 0.6 M) used in the qPCR measurements are listed in Supplementary Table S7. *C*t values were converted to fold changes using the delta-delta-*C*t method (46) in R.

## RESULTS

### Joint modeling identifies high confidence FUS gene expression and splicing targets

To identify transcriptional changes induced by ALS-causing FUS mutations, we performed high depth RNA sequencing on spinal cords from FUS-Δ14 mice, a recently described knockin mouse line carrying a frameshift mutation leading to a complete loss of the nuclear localisation signal (NLS) (Figure 1A and B) (28). As homozygous FUS-Δ14 mice are perinatally lethal, we harvested spinal cord tissue from late-stage embryonic mice (E17.5). In order to directly compare mutation-induced changes to genuine FUS loss of function, we performed similar experiments in parallel using FUS knockout (FUS KO) and littermate control spinal cords at the same developmental time-point (Figure 1C). The FUS KO mutation consists of a ubiquitous deletion from the start to the stop codon of the *FUS* gene that delivers a complete ablation of *FUS* gene expression. We refer to these samples in the manuscript as the ‘Fratta samples’.

To enhance the confidence of our analysis and identify changes relevant across FUS NLS mutations, we took advantage of two publicly available mouse CNS datasets, where ALS-causative mutations in the endogenous *Fus* gene were expressed homozygously, and where FUS KO was used in parallel. The RNA-seq datasets (described in Supplementary Table S1A) were (i) E18.5 brains from FUS-ΔNLS, a model of the R495X mutation that removes the entire NLS (47), along with a GeneTrap FUS-KO that results in a strong knockdown (32), together referred to as the ‘Dupuis samples’; and (ii) ES-derived motor neurons from mice homozygous for the FUS-P517L mutation, corresponding to human P525L (48) that mutates the critical proline residue of the NLS, paired with a FUS-KO (33), together referred to as the ‘Bozzoni samples’. The FUS KO construct used by the Bozzoni lab is a GeneTrap inserted into intron 12 (17), which leads to a partial FUS knockout (Supplementary Figure S1B).

After performing differential expression and differential splicing analyses on each individual dataset, we combined the three datasets and performed two joint analyses for the KO and NLS mutation samples with their respective controls (Figure 1D). Throughout the manuscript, we refer to these two joint models as FUS KO and FUS NLS, respectively. This approach identifies differentially expressed and spliced genes that have a shared direction of effect between the three FUS KO or FUS NLS datasets. Although the three mutations all impair the NLS and subsequent nuclear localization of FUS, some mutations also impact the RGG protein domains upstream of the NLS, which may influence the results. However, using a joint model will prioritize shared effects on expression and splicing and reduce or remove effects specific to each mutation.

### FUS NLS mutations have a loss-of-function effect on gene expression

**Figure 2.**
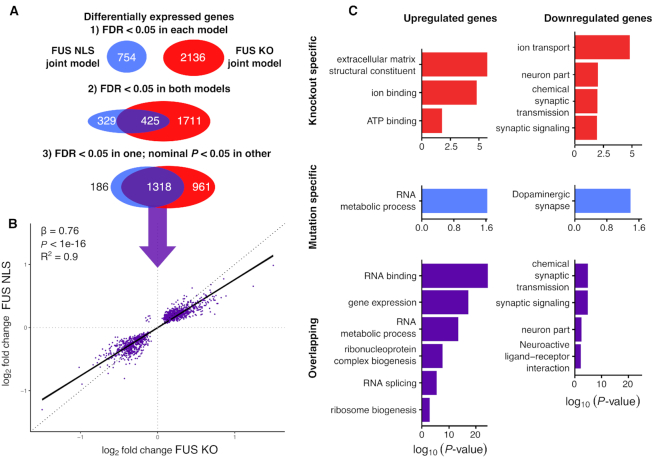
FUS NLS mutations induce a loss of function on expression, with upregulation of RBPs and downregulation of neuronal genes. (**A**) Schematic of the strict and relaxed thresholds for overlapping differentially expressed genes between the two joint models of FUS KO and FUS NLS. (**B**) Plotting the log_2_ fold change for the NLS model against the joint KO model for the overlapping genes only. (**C**) Gene Ontology terms enriched in the three categories of genes split by direction of change.

The joint analysis of differential expression identified 2136 and 754 differentially expressed genes at a false discovery rate (FDR) of 0.05 in FUS KO and FUS NLS, respectively (Figure 2A). The joint analysis identified fewer genes than the sum of all individual datasets, indicating that a large number of genes called as significantly differentially expressed in a single comparison cannot be replicated in the others (Supplementary Table S2A). We then looked for evidence of either a shared or divergent gene expression signal between the FUS KO model and the FUS NLS model. With a conservative threshold for overlap, where a gene must be significant at FDR < 0.05 in both models, we found an overlap of 425 shared genes between FUS KO and FUS NLS, with 329 genes being classified as mutation-specific and 1711 as knockout specific. More permissive overlap criteria, where a gene overlaps if it reaches FDR < 0.05 in one model and an uncorrected *P* < 0.05 in the other, increased the overlap to 1318 genes, reducing the specific genes to 186 in the FUS NLS model, and 961 in KO (Figure 2A). Comparing the direction of changes found for the 1318 overlapping genes between FUS KO and FUS NLS showed that only seven genes are altered in opposing directions, confirming a loss-of-function effect of *Fus* mutations on gene expression (Figure 2B and Supplementary Figure S1A). A linear model fit between the fold changes of the two datasets showed that the effect of FUS NLS on gene expression is 76% that of FUS KO (*β* = 0.76; *P* < 1e-16 *F*-test; *R*^2^ = 0.90) indicating that the magnitude of change is greater in FUS KO than FUS NLS. The relative weakness of NLS mutations compared to knockouts can be explained as NLS mutant FUS can still be detected in the nucleus, albeit at lower amounts (28,49).

### FUS NLS mutations induce synaptic and RNA-binding gene expression changes

Among the most changed genes are the other members of the FET family of RNA-binding proteins to which *Fus* belongs, *Taf15* (Supplementary Figure S1C) and *Ewsr1*. In addition we observed strong changes in *Trove2*, which is downregulated in FUS NLS only and unchanged in FUS KO (Supplementary Figure S1D).

Gene ontology (GO) analyses showed that genes commonly upregulated in both FUS KO and FUS NLS to be enriched in RNA binding, splicing and metabolism terms, and commonly downregulated genes in synaptic and neuronal terms (Figure 2C). knockout-specific and mutation-specific genes were less clearly enriched in specific functions (Figure 2C).

To investigate the relationship between FUS binding and differential expression, we used a FUS iCLIP dataset from embryonic day 18 mouse brain (6). We compared differentially expressed genes to a non-differentially expressed set of genes matched for length and expression levels and compared the proportion of genes in the set bound either by FUS or as a negative control, the splicing factor U2AF65 that should bind to all genes. We found enrichment of FUS binding specifically within downregulated genes. When split into genomic features, we observed this effect to be driven by binding within introns (Supplementary Figure S2).

### FUS NLS mutations induce a splicing loss of function

**Figure 3.**
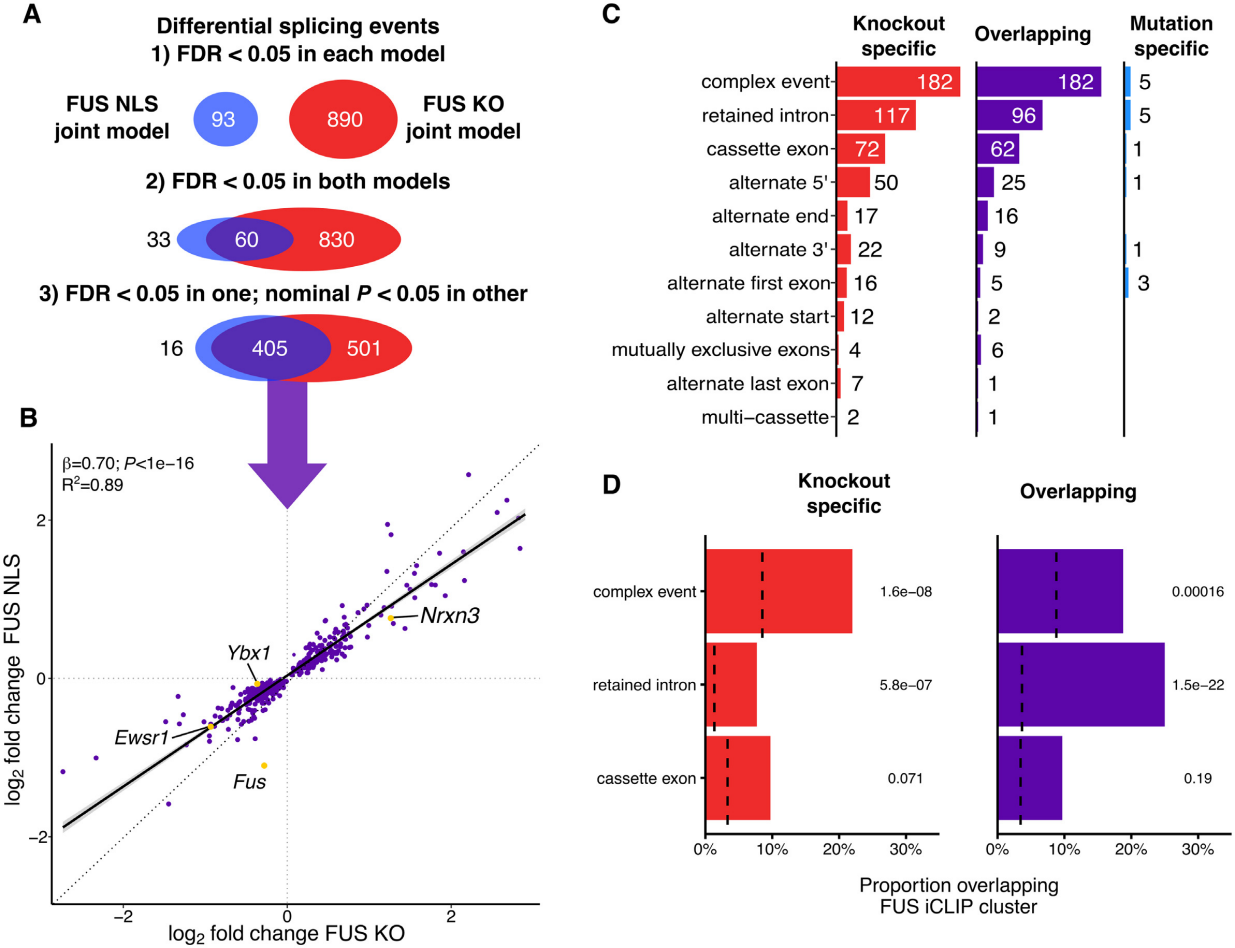
NLS mutant FUS induces a splicing loss of function. (**A**) Schematic of the strict and relaxed thresholds for overlapping differential splicing events between the two joint models of FUS KO and FUS NLS. (**B**) Plotting the log_2_ fold change for the FUS NLS model against FUS KO for the overlapping splicing events. (**C**) Counts of each category of splicing events found in the three sets. (**D**) The proportion of each type of splicing variant in each category that overlap a FUS iCLIP cluster. Background sets of non-regulated splicing events matched for length and wild-type expression are represented by dotted lines. *P*-values from χ^2^ test, corrected for multiple testing with the Bonferroni method.

We used the joint modeling approach to assess the impact of FUS NLS mutations and FUS KO on alternative splicing, including both novel and annotated splicing isoforms. The two joint models found 890 and 93 significant differential splicing events at FDR < 0.05 in FUS KO and FUS NLS (Figure 3A). The joint analysis increased power of detection of splicing changes for both FUS NLS and FUS KO, and identified more events than the sum of the individual analyses (Supplementary Table S2B).

Comparing the joint FUS KO and FUS NLS splicing models, there were 405 overlapping events at the permissive significance threshold used before, with 501 KO specific splicing events and only 16 FUS NLS-specific splicing events (Figure 3A). There were no overlapping splicing events that change in opposing directions, confirming FUS mutations have a loss-of-function effect on splicing. Furthermore, larger fold changes were present in the FUS KO compared to FUS NLS joint models (*β* = 0.7, *P* < 1e-16; *F*-test; *R*^2^ = 0.89), supporting the reduced nuclear localization of the mutant FUS as the main responsible factor for the loss of splicing function.

We then examined sequencing data from heterozygous FUS KO and FUS-Δ14 samples to investigate whether splicing changes had a gene dosage effect. Comparison of splicing events between the heterozygous and homozygous FUS-Δ14 and FUS KO samples found 34 overlapping FUS-Δ14 events and 115 overlapping FUS KO events (Supplementary Figure S3A). Fold changes showed heterozygotes to have a reduced effect size compared to the homozygotes in both FUS-Δ14 (*β* = 0.57; *P* = 1e-11) and FUS KO (*β* = 0.67; *P* < 1e-16), thus demonstrating a gene dosage effect on splicing.

### Intron retention is the most common splicing change induced by FUS mutations

Separating splicing events by type showed a similar distribution between KO-specific and events found in both FUS KO and FUS NLS mutations (‘overlapping events’) as both were dominated by retained introns and complex events (Figure 3C). The latter are difficult to interpret as multiple types of alternative splicing co-occur within the same locus. This can be seen in *Ybx1*, where a retained intron is accompanied by an alternate cassette exon and the splicing of both were altered in both FUS KO and FUS NLS (Supplementary Figure S3B and C). Cassette exons and alternate 5′ and 3′ splice sites were found in all three sets of genes, with alternate 5′ sites appearing at twice the rate of alternate 3′ splice sites. FUS has been shown to interact with the U1 snRNP, which may explain this over-representation (50,51).

*In vivo* and *in vitro* studies of RNA–protein interaction have proposed direct regulation of splicing by FUS through binding to introns (5–8). We used published FUS iCLIP clusters (6) to show that retained introns are strongly enriched for FUS-binding sites, with the strongest enrichment present for the overlapping retained introns (*P* = 4.4e-22; χ^2^ test; Figure 3D). A small but significant enrichment in TDP-43 binding was seen in the same set of events (*P* = 0.027; Supplementary Figure S3D), with no enrichment in U2AF65 binding. Surprisingly, no enrichment of FUS binding was observed in cassette exons, suggesting that these events may not be the direct result of altered FUS expression.

We and others have previously shown that knocking out or knocking down TDP-43 in humans and mice leads to the inclusion of novel exons (so-called ‘cryptic splicing’) (52,53). We then demonstrated that missense mutations in the low-complexity domain of TDP-43 cause the skipping of constitutive included exons, a phenomenon we termed ‘skiptic splicing’ (26). We applied our previously used criteria to classify the cassette exons found to be mis-spliced upon FUS NLS mutation or knockout by their percent spliced in (PSI) values. No exons were classified as skiptic or cryptic, in line with our previously found absence of novel splicing in FUS knockdown data (53).

### FUS-regulated retained introns are enriched in RBP-encoding transcripts and are highly conserved

**Figure 4.**
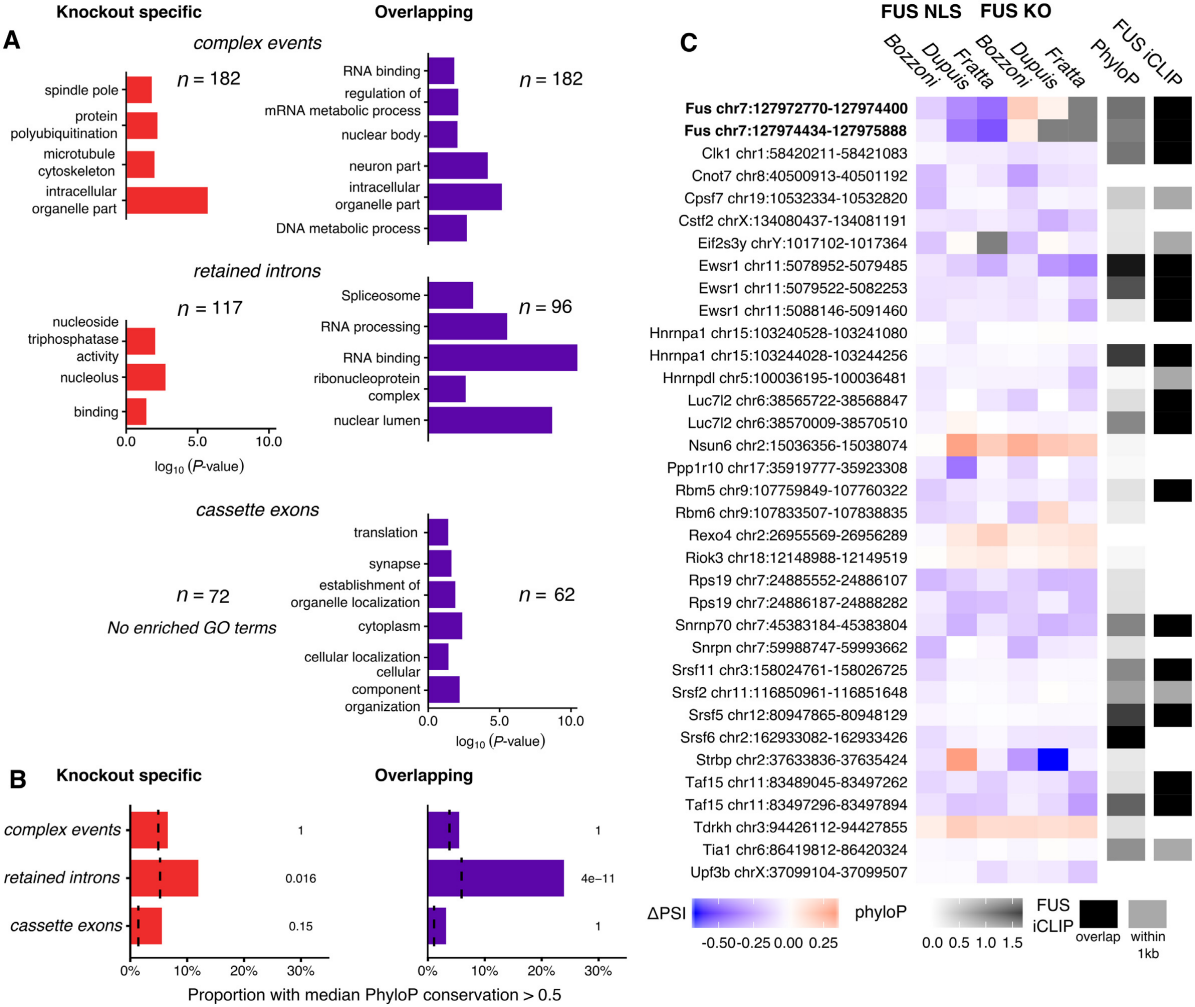
FUS modulates the inclusion of a set of highly conserved RNA-binding protein introns. (**A**) Significantly enriched Gene Ontology terms found in genes split by category and splicing variant type. (**B**) The proportion of each type of splicing event that has a median phyloP conservation score >0.5. Background sets as before. *P*-values from a χ^2^ test, corrected for multiple testing with the Bonferroni method. (**C**) All intron retention events found in the overlapping set found to have an RNA-binding GO term, along with the two FUS introns that are mutation specific. ΔPSI values were calculated for each individual splicing analysis and presented from negative (blue) to positive (red). Events not identifiable in a dataset are colored gray. Median phyloP conservation across each intron coded from 0 (non-conserved; white) to 1.5 (highly conserved; black). Additionally, each intron is noted for the presence of FUS iCLIP cluster overlapping (black) or within 1 kb of either end of the intron (dark gray).

Gene ontology analysis for each category of events showed a clear enrichment in RNA binding and neuronal GO terms in the overlapping splicing events. Specifically, genes with retained introns were often related to RNA binding (Figure 4A). These transcripts include the U1 splicing factor *Snrnp70*, the FET protein family members *Ewsr1* and *Taf15*, and *Fus* itself. Conversely, neuronal GO terms were only enriched in cassette exons.

RBPs often contain intronic sequences that are very highly conserved (54) and have been proposed to be important for their post-transcriptional regulation (55). To test whether the splicing events with confirmed FUS binding show high sequence conservation, we calculated the median phyloP score using the 60-way comparison between mouse and other species for each encompassing intron (42). Sets of events were then tested on the proportion of the set with a median phyloP score >0.5, where a score of 0 is neutral and >1 is highly conserved. Only retained introns were enriched in sequence conservation, and to a greater extent for FUS NLS and FUS KO overlapping (*P* = 1.2e-10) than FUS KO-specific events (*P* = 0.048; Figure 4B). Thirty-five retained intron events are found in genes with RBP-related GO terms. Notably, the direction of change shows these introns to be predominantly decreasing in retention upon FUS KO or NLS mutation. In addition, 20 out of 35 events have a FUS binding iCLIP cluster within 1 kb of the intron region (Figure 4C).

Taken together, these results show that nuclear depletion of FUS through either knockout or NLS mutation leads to a set of splicing changes enriched in conserved intron retention events predominantly affecting RNA-binding proteins. Conversely, cassette exons are not bound by FUS beyond random chance and are flanked by non-conserved introns.

### Modest overlap between differentially expressed and differentially spliced genes

To test whether genes differentially expressed under FUS mutation or knockout are also differentially spliced, we intersected lists of genes from each type of splicing event with the set of 1318 differentially expressed genes in the permissive overlap group. About 18 of 163 genes with at least one complex splicing event were also differentially expressed (*P* = 9.5e-6, Fisher’s exact test), as were 10 of the 83 genes with retained introns (*P* = 1.2e-3) (Supplementary Table S3A). Neither splicing event type had a bias toward up- or down-regulation of expression. Thirty genes in total were both differentially expressed and spliced, including the FET family members *Taf15* and *Ewsr1*, as well as fellow RNA-binding proteins *Srsf6* and *Rbmx* (Supplementary Table S3B). As the majority of differentially spliced genes were not differentially expressed and vice versa, FUS appears to have predominantly separate roles in gene expression and splicing.

### FUS autoregulates through highly conserved retained introns

**Figure 5.**
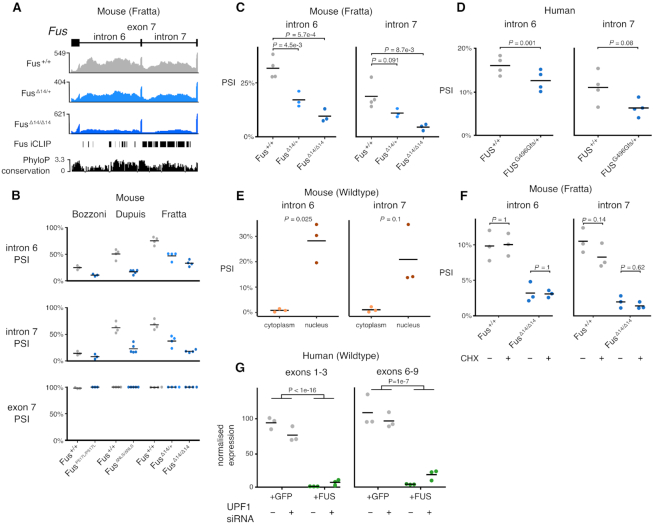
FUS autoregulation occurs through the modulation of an intron retention event leading to transcript nuclear detention. (**A**) FUS introns 6 and 7 are highly conserved and have multiple FUS iCLIP binding peaks. Retention of introns 6 and 7 decreases with increasing dose of FUS-Δ14. RNA-seq coverage for wild-type, FUS-Δ14 heterozygous and FUS-Δ14 homozygous samples are accompanied by FUS iCLIP (6) and phyloP conservation (60 way) tracks. (**B**) Percentage spliced in (PSI) values of intron 6, intron 7 and exon 7 in the three datasets, including the FUS-Δ14 heterozygotes. (**C**) RT-PCR validation of the reduction in introns 6 and 7 inclusion with increasing dose of FUS-Δ14 mutation. Left panel: FUS intron 6; ANOVA genotype *P* = 5.1e-4. Right panel: FUS intron 7; ANOVA genotype *P* = 8.5e-3. Pairwise *t*-tests reported on plot, corrected by Holm method. (**D**) RT-PCR validation of reduced retention of FUS introns 6 and 7 in fibroblasts from a human patient with a FUS G496Gfs mutation (*n* = 1) compared to a healthy control (*n* = 1). RT-PCR repeated in triplicate for each sample. ANOVA genotype *P* = 0.001; intron 7 ANOVA genotype *P* = 0.08 (**E**) RT-PCR on FUS introns 6 and 7 on nuclear and cytoplasmic RNA fractions. Intron 6 *t*-test *P* = 0.025; Intron 7 *t*-test *P* = 0.1. (**F**) Translation blocked with cycloheximide (CHX) to observe whether the intron retention transcript is sensitive to nonsense-mediated decay. Left panel: FUS intron 6 retention is not altered with CHX treatment. ANOVA treatment *P* = 0.96; genotype *P* = 5.7e-5; interaction *P* = 0.86. Right panel: FUS intron 7 retention is unchanged by CHX treatment. ANOVA treatment *P* = 0.10; genotype *P* = 7.9e-6; interaction *P* = 0.1. Pairwise *t*-tests reported on plot, corrected by Holm method. (**G**) Reduced endogenous FUS RNA levels in HeLa cells expressing codon-optimized FUS compared to HeLa cells expressing a GFP control, as measured by qPCR. This reduction was mostly unaffected by the siRNA depletion of UPF1.

The joint splicing analyses found two retained introns (introns 6 and 7) in the *Fus* transcript to be less retained in FUS NLS mutants. Both introns are highly conserved across mammalian species and contain multiple FUS iCLIP peaks in both mouse and human (Figure 5A). As retention of these introns decreased in the presence of FUS mutations, we hypothesized that their retention could have a regulatory function on FUS expression. Numerous RBPs regulate their expression by binding their own transcript (24,56–58) so that when protein levels are high, increased binding of the pre-mRNA shifts alternative splicing toward the production of an untranslated isoform, either degraded by nonsense-mediated mRNA decay (NMD) or by detaining the transcript in the nucleus to avoid translation in the cytoplasm (59–62). The FUS intron 6/7 region is the putative locus of FUS autoregulation through the skipping of exon 7 that causes a frameshift, producing an NMD-sensitive transcript (25). However, when examining RNA-seq junction coverage of the FUS gene in all our mouse datasets, we failed to observe skipping of exon 7 in any sample (Figure 5B). Instead, the retention of both introns 6 and 7 decreased in the presence of FUS mutations in all three datasets, despite the baseline level of intron retention in wild-type samples being highly variable between datasets. Heterozygous FUS-Δ14 mice also showed a significant reduction in intron retention, albeit less than the homozygotes, demonstrating a dose-dependent response. We validated our findings using RT-PCR and confirmed that intron retention decreases in a mutation dose-dependent manner (Figure 5C; intron 6 *P* = 5.1e-4; intron 7 *P* = 8.5e-3; ANOVA; Supplementary Figure S4). We failed to detect a band corresponding to the skipping of exon 7 in any sample.

### FUS autoregulation is conserved in human cells

We then tested whether the same phenomenon of reduced intron retention occurs in human cells, and used primary fibroblasts from a patient carrying the ALS-causative heterozygous FUS mutation G496Gfs that induces a strong cytoplasmic FUS mislocalization through a frameshift that removes the NLS (28). RT-PCR showed a decrease in both introns 6 and 7 retention relative to a FUS wild-type human sample (Figure 5D; intron 6 *P* = 0.001; intron 7 *P* = 0.08; ANOVA; Supplementary Figure S5). These changes were smaller than those observed in homozygous mice, and more similar to the heterozygous mice. As with the mouse samples, no change in exon 7 splicing was observed.

### FUS intron 6/7 retention determines transcript nuclear detention

As retained intron transcripts can accumulate in the nucleus (62), we performed cellular fractionation on wild-type mouse ventral horn spinal cord cultures and performed RT-PCR to assess the level of retention of introns 6 and 7 in the nucleus and cytoplasm. We observed retention of either intron only in the nuclear fraction (Figure 5E and Supplementary Figure S6). These data are in agreement with a recent study using the APEX-seq method to label RNA in different cellular compartments (63), which found FUS introns 6 and 7 to be enriched in the nucleus in human cells. FUS introns 6/7 also contain numerous premature stop codons suggesting they may undergo nonsense-mediated decay (NMD), which may explain the absence of intron retention transcripts in the cytoplasm. In order to understand whether *FUS* intron-retention transcripts are degraded in the cytoplasm or are instead detained in the nucleus, we tested their sensitivity to NMD. Following incubation with cycloheximide (CHX) to inhibit translation and therefore block NMD (64), we observed no change in intron retention in either wild-type or FUS-Δ14 homozygous cells (Figure 5F), despite observing a robust inhibition of NMD when looking at a known event in Srsf7 (Supplementary Figure S7). Similar results were obtained in human cells when UPF1, an essential NMD factor, was knocked down using siRNA (Figure 5G and Supplementary Figure S8), showing only a modest effect on FUS mRNA levels.

In order to further validate FUS autoregulation, we investigated the effect of FUS overexpression. FUS overexpression has previously been shown to downregulate endogenous *Fus* transcripts (20,25,65). However, the presence of an overexpression plasmid makes it difficult to evaluate the expression of endogenous *FUS*. To circumvent this, we generated stably transduced HeLa cell lines that constitutively overexpress *FUS* cDNA using alternative codons, allowing us to design qPCR primers that selectively amplify endogenous *FUS*, but not the overexpressed version. We investigated the effects of FUS overexpression on endogenous *FUS* expression using qPCR. As expected, FUS overexpressing cells showed a strong downregulation of endogenous *FUS* mRNA compared to cells expressing a GFP construct (Exons 1–3 *P* < 1e-16; Exons 6–9 *P* = 1e-7; ANOVA; Figure 5G).

Taken together, these experiments show that *FUS* intron retention is NMD-insensitive. We therefore suggest that FUS regulates its own expression through the retention of introns 6 and 7. This transcript is detained in the nucleus, reducing the amount of cytoplasmic FUS available for translation (Figure 7).

### FUS intron retention is co-regulated by TDP-43 and altered in other ALS models

FUS shares RNA targets with another ALS-associated RNA-binding protein, TDP-43 (5). Despite FUS iCLIP peaks being present throughout the *Tardbp* gene, neither FUS knockout nor NLS mutation altered *Tardbp* expression or splicing.

**Figure 6.**
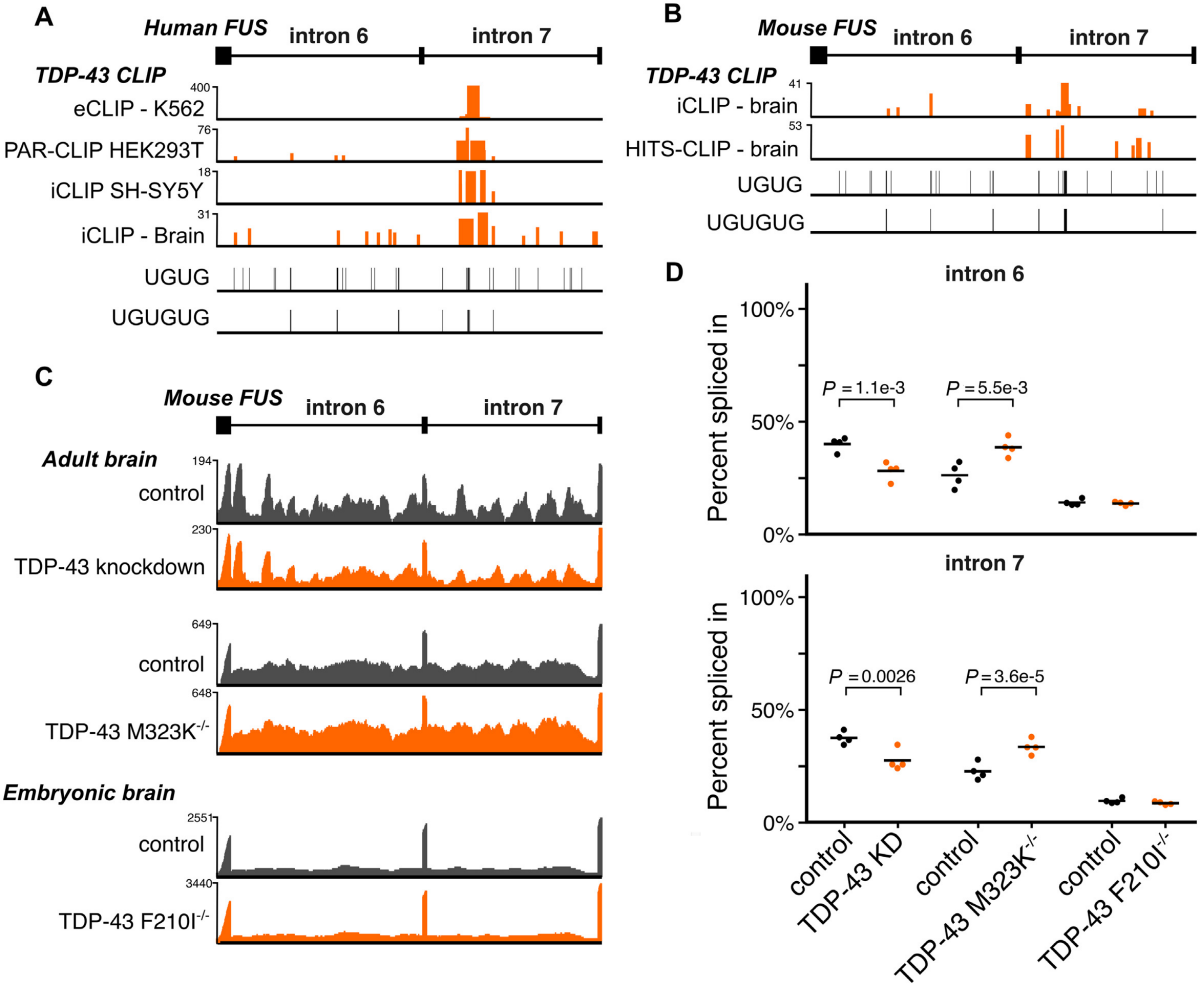
TDP-43 co-regulates FUS intron retention. (**A**) Mouse TDP-43 cross-linking and immunoprecipitation (CLIP) data overlap a TG-rich section of *Fus* intron 7. (**B**) Human TDP-43 CLIP data overlap within *FUS* intron 7 in a section rich with TG sequence. (**C**) RNA-seq traces of representative samples demonstrate decreased *Fus* intron retention in TDP-43 knockdown and increased retention in TDP-43 M323K mutation, both in adult mouse brain. No effect is seen with the RNA-binding mutant F210I in embryonic mouse brain. *Y-*axis of each trace refers to the maximum read depth. (**D**) Percentage spliced in quantification from each TDP-43 dataset of introns 6 and 7 retention. *P*-values are presented from splicing analysis on each dataset.

However, using TDP-43 CLIP data from human and mouse collected by the POSTAR database (36), we identified a conserved TDP-43 binding site within *FUS* intron 7, ∼400 nucleotides downstream of the 5′ splice site in a UG-rich region conserved between mouse and humans (Figure 6A and B). UG dinucleotides are the known binding motif of TDP-43 (66). To test whether TDP-43 is also involved in regulating the retention of FUS introns 6 and 7, we re-analyzed RNA-seq data where TDP-43 was knocked down in adult mice with an antisense oligonucleotide (4) as well as adult mice homozygous for a C-terminal TDP-43 mutation (M323K) that we previously showed to cause a gain of splicing function (26). TDP-43 knockdown caused a reduction in FUS intron retention, similar to FUS NLS mutations (Figure 6C). Conversely, TDP-43 M323K mice had an increase in FUS intron retention relative to wild-type. We observed no changes in FUS intron retention in embryonic mice homozygous for a splicing-null mutation (F210I) (26), suggesting a developmental component to TDP-43 cross-regulation of FUS. FUS introns 6 and 7 are also bound by fellow FET family members TAF15 and EWSR1 in human and mouse (Supplementary Figure S9).

A previous study differentiated human motor neurons from induced pluripotent stem cells (iPSC) with and without mutations in VCP, an ALS-causative gene that induces TDP-43 pathology (34,67). The authors observed a set of retained intron events that change earlier in differentiation in the VCP mutant cells compared to the wild-type. These changes occurred primarily in RBPs, suggesting a perturbation in splicing factor networks during development. To compare the FUS-regulated murine splicing events with those found in VCP mutant human cells, we overlapped the 143 human genes found to have intron retention events with the set of 219 mouse genes with either intron retention or complex events in both FUS NLS and FUS KO and found an overlap of 12 genes (*P* = 1e-5, Fisher’s exact test; Supplementary Table S4). Remarkably, this included FUS itself. Re-analysis of published RNA-seq data from (34) demonstrated that the retention of FUS introns 6 and 7 is increased specifically in the transition between iPSC and neural precursor cell (NPC) stage in the VCP mutants (Supplementary Figure S10A). Neither *FUS* nor *TARDBP* was differentially expressed at any stage (Supplementary Figure S11).

The same study also found intron retention changes in another ALS model, SOD1 A4V mutations in iPSC-derived motor neurons (35). Re-analysis of the same RNA sequencing data showed a robust increase in FUS intron retention in the presence of SOD1 mutations (Supplementary Figure S10B). Again, neither *FUS* nor *TARDBP* was found to be differentially expressed in this dataset (Supplementary Figure S11).

At last, we asked whether FUS intron retention was altered in human patients with ALS or FTD. We re-analysed two human post-mortem brain datasets: one from FTD patients with either Tau (FTD-Tau) or TDP-43 (FTD-TDP) pathology (37) and the second from ALS patients with either C9orf72 mutations (c9ALS) or without known mutations (sALS) where both groups should have TDP-43 pathology (38). Both datasets sampled frontal cortex and cerebellum tissue from patients along with non-neurological disease controls. The cerebellum does not exhibit any TDP-43 pathology in either disease (68). Therefore, any TDP-43-related splicing changes should be restricted to the frontal cortex. These two datasets allow us to test whether FUS intron retention changes occur only in patients and tissues that had pathological TDP-43 mislocalization.

The two datasets had very different overall levels of FUS intron retention with ∼75% retention of both FUS introns in the FTD dataset but levels closer to 20% in the ALS dataset (Supplementary Figure S12), despite the two RNA-seq datasets being prepared similarly (Supplementary Table S8). No significant difference in intron retention levels or in exon 7 skipping between disease and control was observed in either dataset. As TDP-43 mislocalization occurs only in a minority of cells, a larger sample size or a cell-type specific assay may be needed to reveal changes in FUS splicing in post-mortem human brain samples.

Taken all together, these findings suggest that FUS regulation may be altered in the presence of ALS-causative mutations in other genes.

## DISCUSSION

FUS is a central player in ALS biology and its role in RNA metabolism has been intensively studied, but how disease-causing mutations impact upon RNA processing is still being determined. One limitation has been that FUS, like many other RBPs, is extremely sensitive to gene dosage. Therefore, commonly used models, where mutant FUS is overexpressed, cannot disentangle the effects of overexpression from those of the mutation. We and others have generated FUS-ALS models where mutations were inserted into the endogenous gene to observe the impact on gene expression and splicing. In order to specifically investigate splicing changes, we generated high depth sequencing data from spinal cords of our mutant FUS mice, alongside FUS knockout samples, to compare mutant-induced changes to a pure loss of function. In order to identify with high confidence changes relevant to multiple FUS-ALS models we performed a joint analysis, combining our data with other publicly available datasets where endogenous *Fus* mutations had been studied in parallel to samples from knockout tissue. The sequencing conditions differed between datasets (Supplementary Table S1B), with some more suited for expression analysis rather than for splicing analysis. Nonetheless, the joint analysis model proved to be extremely powerful and allowed us to identify more splicing changes than using any single dataset independently. Furthermore, this approach limits artifactual findings from individual datasets and allowed us to define a comprehensive high-confidence list of both expression and splicing targets induced by ALS-FUS mutations and FUS loss.

The comparisons between the joint analysis of FUS NLS mutations and FUS knockout show that FUS mutations have a loss-of-function effect both on expression and splicing. This is a key difference from mutations in TDP-43, the other major RBP implicated in ALS, where specific mutations lead to gain of splicing function (26,27). The fact that the changes appear weaker in NLS mutations compared to FUS knockout is compatible with mutant FUS still being found at low levels in the nucleus of all the analyzed mutants, and supports a dosage-dependent nuclear loss of nuclear function.

The high depth and relatively long reads (150 bp) of our sequencing data allowed us to conduct an unbiased analysis of splicing, which highlighted intron retention and complex splicing events to be the most frequently altered class of changes, whilst cassette exon events, which had been previously described by using a targeted approach (32), are less abundant. Interestingly, when we assessed the association of FUS binding to different splicing events using CLIP, FUS was linked directly to intron retention, but not to cassette exon splicing, suggesting the latter category could be due to downstream or indirect effects.

Intriguingly, the FUS transcript features among the strongest intron retention changes induced by FUS mutations, with the mutation inducing a reduction in retention of introns 6 and 7. This led us to consider the possibility that FUS intron 6/7 retention had a biological function. This region has been previously suggested to be involved in FUS autoregulation through alternative splicing of exon 7 (25), but we were unable to observe this at significant levels in any of the datasets we analyzed in both human and mouse. The two introns are highly conserved across species, and FUS iCLIP data showed widespread FUS binding across both introns, supporting their retention as a putative autoregulatory mechanism.

**Figure 7.**
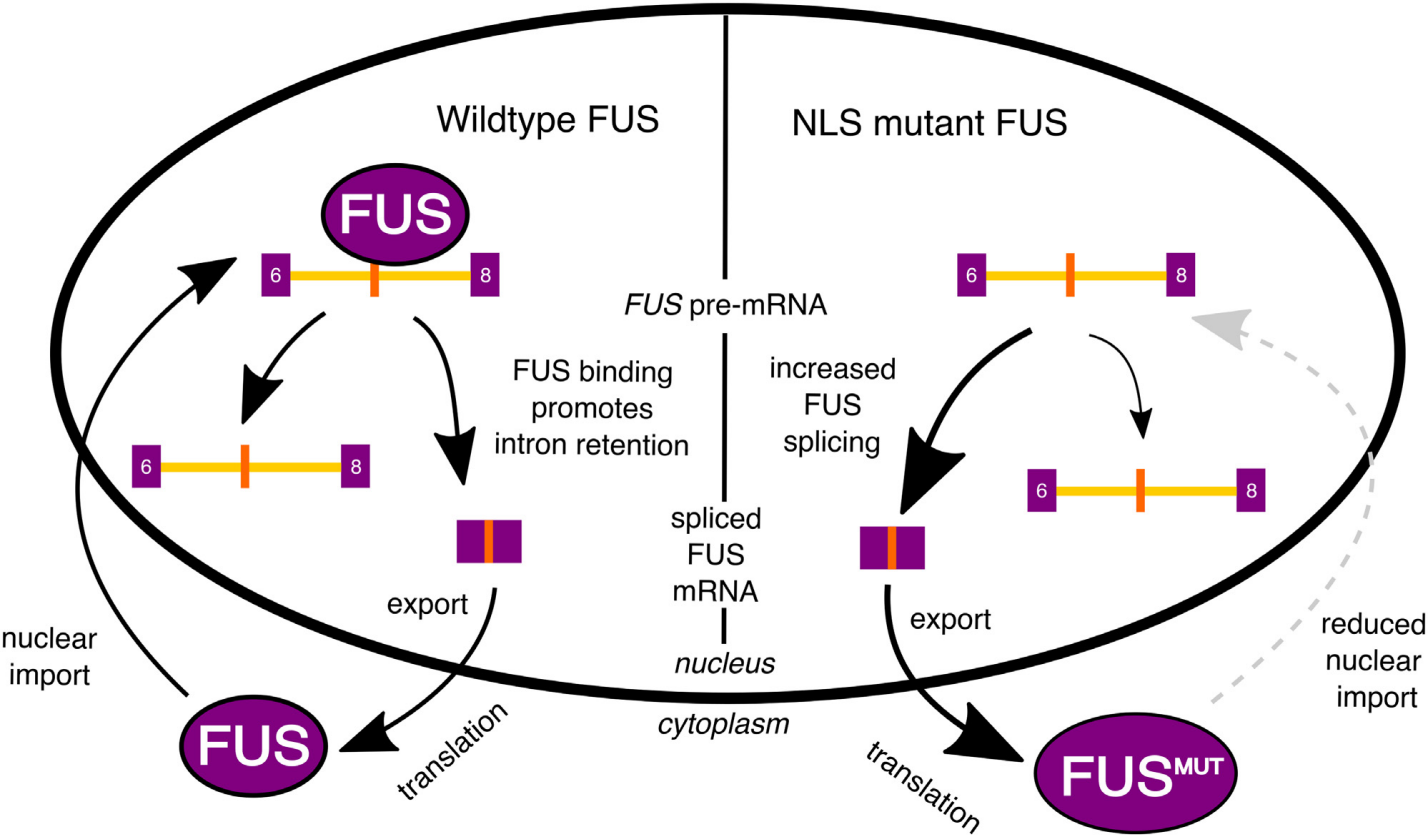
Schematic of proposed FUS autoregulation mechanism. In wild-type cells, FUS protein shuttles between the nucleus and cytoplasm. FUS binding within *FUS* introns 6 and 7 promotes their retention. The intron retention transcript is restricted to the nucleus, reducing the amount of cytoplasmic *FUS* mRNA available for translation. In conditions of low FUS protein, intron retention will be reduced and cytoplasmic *FUS* transcript will be increased. In contrast, in cells with FUS NLS mutations, mutant FUS is not transported to the nucleus as effectively. This reduces the ability of FUS protein to regulate FUS mRNA production through intron retention. This could lead to a vicious cycle of ever-increasing FUS protein in the cytoplasm, which may have toxic effects.

Comparing nuclear and cytoplasmic fractions we found that FUS intron 6/7 retention is preferentially localized to the nucleus (Figure 5E), and experiments in both human and mouse cells found the retained intron transcripts, which are predicted to undergo NMD, to be NMD-insensitive (Figure 5F–G), suggesting they are not degraded in the cytoplasm, but are instead detained in the nucleus. Nuclear detention is a recognized mechanism to control gene expression (62) and has been described to be important in the regulation mechanism of other RNA-binding proteins through exosome-dependent nuclear RNA degradation (69). We therefore propose a new autoregulation mechanism for FUS, whereby the level of nuclear FUS protein is buffered by its binding to the FUS mRNA. Increased nuclear FUS would increase the retention of *FUS* introns 6 and 7, leading to transcript nuclear detention and reduced FUS protein levels in a negative feedback loop (Figure 7). Transcripts with detained introns have been characterized as either ‘reservoirs’ of RNA to be spliced at a later time or ‘dead ends’, where the transcript is subsequently degraded (70). Further work will be needed to determine the fate of the intron retained FUS transcript.

The discrepancy between our results and those of Zhou *et al.* (25) could be explained by FUS autoregulation having two independent mechanisms, using either detained introns or NMD-sensitive exon skipping depending on the cell type and developmental time-point. This would be analogous to TDP-43, where both the nuclear retention of a long 3′ UTR transcript as well as the production of an NMD-sensitive transcript through 3′ UTR splicing have both been observed (24,71). Further work could elucidate that autoregulatory mechanism is favored in different cell types.

Genes changed both at the splicing and expression level by *Fus* mutations are enriched in ‘RNA metabolism’ GO terms, supporting a secondary effect on splicing of other RBPs. Furthermore, the altered intron retention events are specifically enriched for ‘RNA metabolism’ suggesting that also a cross-regulation among RBPs may occur through this mechanism, which is compatible with the growing evidence of RBPs functioning as a sophisticated regulatory network (60,72–74). This raises the question as to what other proteins contribute to the regulation of FUS levels, and whether other ALS-linked proteins could play a role. FUS binds and regulates the levels and splicing of EWSR1 and TAF15, both associated with ALS through rare familial mutations (75,76) and both EWSR1 and TAF15 bind to *Fus* introns 6/7 (Supplementary Figure S9). Although FUS depletion leads to an upregulation of TAF15, reducing TAF15 has no effect on FUS expression (77). Intriguingly, we found TDP-43 to bind reproducibly to intron 7 in both human and mouse, and that in adult mice TDP-43 knockdown induces a significant decrease in retention of both introns, whilst the opposite was found in the presence of gain-of-function TDP-43 mutations. No changes were found in an embryonic dataset from mice where TDP-43 has a decreased RNA-binding capacity. This observation could be due to different co-regulation patterns occurring across developmental stages (78,79). Our findings support a role for TDP-43 in regulating FUS through the same post-transcriptional mechanism by which FUS regulates itself.

*FUS* intron retention is also altered in the presence of both VCP and SOD1 mutations, two ALS-related genes that are not RBPs but have recently been linked to alterations in intron retention (34). Of note, whilst VCP mutations lead to typical TDP-43 pathology, SOD1 mutant cases do not show TDP-43 mislocalization, but rather cytoplasmic inclusions of misfolded SOD1. Our findings of FUS regulation being altered in VCP and SOD1 cases do not show a direct link between these proteins and FUS regulation, but rather support FUS dysregulation being present across a wide range of ALS cases, in line with recent findings (80). Future targeted studies could more sensitively assess changes in FUS regulation across a variety of conditions. Such studies could make use of a minigene incorporating FUS introns 6 and 7.

Finally, we re-analyzed transcriptome data from human post-mortem brain in ALS and FTLD to test whether TDP-43 pathology, seen in both diseases, would be reflected by changes in FUS splicing. The fact we could not observe any changes may be due to the high variability of post-mortem disease brain tissue and the fact that TDP-43 mislocalization occurs in a minority of cells, compared to more homogeneous cell lines or animal models. Larger cohorts of post-mortem brains or experiments isolating cells with TDP-43 pathology such as (81) will be required to fully explore this question.

In conclusion, we have found that FUS mutations induce a loss of splicing function, particularly affecting intron retention events in other RBPs. We show that an intron retention event in the *FUS* transcript is a mechanism for its autoregulation and is modified not only by mutations in FUS, but also by ALS-causative mutations in TDP-43, VCP and SOD1. Altered regulation of FUS can contribute to neurotoxicity. Elucidating how this occurs and how other ALS proteins play a role in this mechanism advances our understanding of the ALS pathogenic cascade.

## DATA AVAILABILITY

RNA-seq data for the FUS-Δ14 mutant and FUS KO homozygous and heterozygous spinal cord samples with their respective wild-type littermate controls has been deposited in the Gene Expression Omnibus (GEO) with accession number GSE147288.

## Supplementary Material

gkaa410_Supplemental_Files

## Notes

Present address: Jack Humphrey, Ronald M. Loeb Center for Alzheimer’s Disease, Department of Neuroscience and Friedman Brain Institute, Icahn School of Medicine at Mount Sinai, New York, NY 10129, USA.
