# Supplementary material for: FUS ALS-causative mutations impair FUS autoregulation and splicing factor networks through intron retention
Source: Nucleic Acids Res. 2020 Jun 1;48(12):6889–905. doi: 10.1093/nar/gkaa410 (PMC7337901; doi:10.1093/nar/gkaa410)
Supplement: gkaa410_Supplemental_Files [file gkaa410_supplemental_files.zip › FUS_AUTOREG_supplementary_material.pdf]

FUS ALS-causative mutations impair FUS autoregulation and splicing factor networks through intron retention

Jack Humphrey[1,2,4*#], Nicol Birsa[1,2], Carmelo Milioto[1,2], Martha McLaughlin[1], Agnieszka M. Ule[1], David Robaldo[1,2], Andrea B Eberle[3], Rahel Kräuchi[3], Matthew Bentham[1], Anna-Leigh Brown[1], Seth Jarvis[1,2,4], Cristian Bodo[1], Maria Giovanna Garone[5], Anny Devoy[1,2,6], Alessandro Rosa[5,7], Irene Bozzoni[5,7], Elizabeth MC Fisher[1], Oliver Mühlemann[3], Giampietro Schiavo[1,2,8], Marc-David Ruepp[2,6], Adrian M Isaacs[1,2], Vincent Plagnol[4], Pietro Fratta[1*]

*1. UCL Queen Square Institute of Neurology, University College London, London WC1N 6BG, UK. 2. UK Dementia Research Institute, London, UK 3. Department of Chemistry and Biochemistry, University of Bern, Freiestrasse 3, 3012 Bern, Switzerland 4. UCL Genetics Institute, University College London, London WC1E 6BT, UK. 5. Sapienza University of Rome, Rome, IT. 6. Maurice Wohl Clinical Neuroscience Institute, King's College London, London, UK. 7. Center for Life Nano Science, Istituto Italiano di Tecnologia, Rome, IT. 8. Discoveries Centre for Regenerative and Precision Medicine, University College London Campus, London WC1N 3BG, UK. * Corresponding authors # Current address: Ronald M. Loeb Center for Alzheimer's Disease, Department of Neuroscience and Friedman Brain Institute, Icahn School of Medicine at Mount Sinai, New York, NY, 10129, USA.*

Supplementary Material

Supplementary Data 2

Supplementary Tables 4

Supplementary Table 1A: The three FUS mouse datasets used in this study. 4
Supplementary Table 1B: Characteristics of the three sequencing datasets. 4
Supplementary Table 2A: The results of the individual and joint gene expression analyses. 5
Supplementary Table 2B: The results of the individual and joint splicing analyses. 5
Supplementary Table 3A: Overlap between differential expression (DE) and splicing (DS) in both models, split by type of splicing event 6
Supplementary Table 3B: Genes that are both differentially spliced and differentially expressed in both models. 7
Supplementary Table 4: Genes overlapping with Luisier et al: 8
Supplementary Table 5: Primer sequences used for RT-PCR in mouse 9
Supplementary Table 6: Primer sequences used for RT-PCR in human 10
Supplementary Table 7: primers used for RT-qPCR in human cells 11
Supplementary Table 8: Characteristics of other RNA-seq datasets used 12

Supplementary Figures 13

Supplementary Figure 1: Overlapping gene expression models show concordant effects of FUS KO and FUS NLS mutations 13
Supplementary Figure 2: Downregulated genes are enriched in FUS iCLIP clusters 14
Supplementary Figure 3: Accompaniment to splicing analyses 15
Supplementary Figure 4: FUS, EWSR1, TAF15 and TDP-43 bind FUS introns 6 and 7 in human and mouse 17
Supplementary Figure 5: RT-PCR of FUS intron retention in mouse 18
Supplementary Figure 6: RT-PCR of FUS intron retention in human samples 19
Supplementary Figure 7: Nuclear and cytoplasmic fractionation 20
Supplementary Figure 8: Cycloheximide inhibition experiments 22
Supplementary Figure 9: FUS overexpression qPCR and UPF1 knockdown 23
Supplementary Figure 10: FUS intron retention is dysregulated in different ALS models 24
Supplementary Figure 11: FUS and TARDBP expression changes in VCP and SOD1 mutations 25
Supplementary Figure 12: FUS intron retention in human ALS and FTD post-mortem brain 26

Supplementary Data

1. *supplementary_data_differential_expression.xlsx* - an excel file containing sheets for the full results of DESeq2 differential gene expression in each individual FUS mouse line against its controls, a description of the columns used, and the joint model results containing log_2 fold changes, P-values and adjusted P values for each gene in the two models and the overlap classifications.
2. *expression_GO_full.tsv* - all GO terms found in each category of differentially expressed genes
3. *supplementary_data_differential_splicing.xlsx* - an excel file containing sheets for the significant (FDR < 0.05) splicing results for an individual FUS mouse line against its controls, a description of the columns used, and the joint model results containing log_2 fold changes for each joint model, mean percent spliced in (PSI) values and delta PSI for both joint models and each individual comparison, iCLIP and PhyloP results used in the manuscript, and the overlap classifications.
4. *splicing_GO_full.tsv* - all GO terms found in each category of splicing event

Supplementary Tables

Supplementary Table 1A: The three FUS mouse datasets used in this study.

mESC - mouse embryonic stem cell

Dataset	Tissue	Controls	Age	Knockout (KO)	Mutation (MUT)
Bozzoni (Capauto et al. 2018)	Motor neurons cultured from mESCs	Shared	-	Gene trap in exon 12 (Hicks et al. 2000)	P517L knock-in, corresponding to human P525L
Dupuis (Scekic-Zahirovic et al. 2016)	Whole brain	Separate	E18.5	Gene trap in intron 1 (Scekic-Zahirovic et al. 2016)	Stop codon after exon 14 (ΔNLS)
Fratta	Spinal cord	Separate	E17.5	Gene trap in intron 1	FUS-Δ14 - splice site mutation

Supplementary Table 1B: Characteristics of the three sequencing datasets.

Dataset	Replicates per condition	Library type	Mapped reads (millions)	Read type	Accession (SRA)
Bozzoni	3	Total RNA	34-52	2 x 100bp	SRP111475
Dupuis	4-5	polyA+	15-25	1 x 50bp	SRP070906
Fratta	4	Total RNA	52-65	2 x 150bp	PRJNA528969

Supplementary Table 2A: The results of the individual and joint gene expression analyses.

Numbers refer to the number of genes found to be differentially expressed at FDR < 0.05. Strict overlap between joint models refers to genes with FDR < 0.05 in both models. Relaxed overlap refers to genes with FDR < 0.05 in one model and P < 0.05 in the other.

	**Bozzoni MUT**	**Dupuis MUT**	**Fratta MUT**	**Bozzoni KO**	**Dupuis KO**	**Fratta KO**
Individual analysis	19	1552	88	100	2916	151
Joint analysis	**754**			**2136**		
Overlapping joint model	5	368	57	51	1007	114
Unique to dataset	14	1184	31	49	1909	37
Overlap (strict)	329		**425**		1711	
Overlap (relaxed)	186		**1318**		961	

Supplementary Table 2B: The results of the individual and joint splicing analyses.

Numbers refer to number of splicing events found to be differentially used at FDR < 0.05

	**Bozzoni MUT**	**Dupuis MUT**	**Fratta MUT**	**Bozzoni KO**	**Dupuis KO**	**Fratta KO**
Total (FDR < 0.05)	31	1	56	211	46	230
Joint model	**93**			**890**		
Overlapping joint model	7	1	30	143	38	169
Unique to dataset	21	0	11	67	8	58
Overlap (strict)	33		**60**		830	
Overlap (relaxed)	16		**405**		501	

Supplementary Table 3A: Overlap between differential expression (DE) and splicing (DS) in both models, split by type of splicing event

P-values from Fisher exact test, adjusted by Bonferroni correction.

event type	DE genes	DS genes	overlap	adjusted P
**Compex**	1318	163	18	9.5E-06
**Retained intron**	1318	83	10	0.0012
**Multi-exon skipping**	1318	1	1	0.31
**Alt. 5′**	1318	25	3	0.35
**Alt. 3′**	1318	9	0	1
**Alt. end**	1318	9	1	1
**Alt. first exon**	1318	4	0	1
**Alt. last exon**	1318	1	0	1
**Alt. start**	1318	1	0	1
**Mut. exclusive exon**	1318	3	0	1
**Cassette exon**	1318	60	3	1

Supplementary Table 3B: Genes that are both differentially spliced and differentially expressed in both models.

Full differential expression and splicing tables are present in supplementary data files.

Gene	log_2 Fold Change (FUS KO)	P-value (FUS KO)	log_2 Fold Change (FUS NLS)	P-value (FUS NLS)	Splicing event types
Taf15	0.589	3E-22	0.44	1.4E-12	retained intron+complex
Rap1gds1	-0.352	3.4E-13	-0.23	2.9E-06	complex
Ncapd3	0.244	1.4E-09	0.19	6.1E-06	alt. 5’
Rmnd1	0.338	4E-08	0.15	1.5E-02	complex
Clgn	0.356	2.1E-06	0.22	4.6E-03	cassette exon
Rbmx	-0.259	3.7E-06	-0.19	1.1E-03	complex
Sin3b	0.332	4E-06	0.26	3.4E-04	alt. end
Gm6483	-0.376	4.2E-06	-0.41	6.2E-07	complex
Tpm1	-0.174	1.6E-05	-0.10	1.1E-02	complex
Ewsr1	0.162	2.1E-05	0.11	3.7E-03	retained intron+complex
Wdr7	-0.131	3.1E-05	-0.11	5.5E-04	complex
5730507C01Rik	-0.448	8.2E-05	-0.25	3.2E-02	retained intron
Nrbp1	0.173	1.6E-04	0.23	9.9E-07	retained intron
Hpf1	-0.248	1.7E-04	-0.18	7.5E-03	complex
Cpt1c	0.318	2E-04	0.19	2.8E-02	cassette exon
Aifm1	0.201	2.1E-04	0.16	3.6E-03	complex
Ttc3	-0.219	2.4E-04	-0.20	1.1E-03	complex+retained intron+alt. 5’
Phf14	-0.250	4.6E-04	-0.20	5.3E-03	complex
Cbx1	0.161	4.9E-04	0.10	3E-02	complex+retained intron
Xpa	0.229	5.5E-04	0.16	1.9E-02	complex
Dnm1l	-0.228	7.8E-04	-0.18	8.7E-03	complex
Mok	0.260	8E-04	0.18	2.3E-02	multi skipped exon
Akap6	-0.135	1.1E-03	-0.15	2.7E-04	complex
Hspa4l	-0.204	1.1E-03	-0.21	8.3E-04	retained intron
Eml4	-0.142	1.2E-03	-0.13	3.6E-03	complex+retained intron
Fibp	0.248	1.9E-03	0.16	4.5E-02	alt 5’
Srsf6	-0.208	3.2E-03	-0.15	3.6E-02	retained intron
1700020I14Rik	-0.232	3.3E-03	-0.31	1.2E-04	complex
Ubn1	0.097	1.5E-02	0.17	3.7E-05	retained intron
H13	0.127	3.5E-02	0.23	1.4E-04	cassette exon

Supplementary Table 4: Genes overlapping with Luisier et al:

gene name	coords (mm10)	event length/ bp	variant type	nearest FUS iCLIP cluster/kb	median phyloP
Atp13a3	chr16:30357274-30361420	1446	complex	0.54	0.234
Ccdc88a	chr11:29494093-29499335	5242	complex	66	0.439
Cdc16	chr8:13767587-13768561	974	retained intron	158	0.244
Fbxl5	chr5:43759825-43760707	882	retained intron	646	0.002
Fus	chr7:127972770-127974400	1630	retained intron	0	1.206
Hnrnpdl	chr5:100036195-100036481	286	retained intron	0.31	0.061
Mfn1	chr3:32562893-32563012	119	retained intron	0	0.255
Ncor1	chr11:62401267-62403799	2532	complex	2.80	0.878
Papola	chr12:105829277-105834710	5433	complex	7.32	0.234
Rbm6	chr9:107833507-107838835	5328	retained intron	13.8	0.150
Srsf5	chr12:80947865-80948129	264	retained intron	0	1.712
Tcerg1	chr18:42550108-42551110	1002	retained intron	25.5	0.428

Supplementary Table 5: Primer sequences used for RT-PCR in mouse

Name	Orientation	Target	Sequence (5'- 3')
mFUS-Exon 6-F3	Forward	Fus exon 6	GTTATGGCAATCAGGACCAGAG
mFUS-Intron 6-R2	Reverse	Fus intron 6	TTGGCTCCCAAGTTCTCACA
mFUS-Intron 7-F1	Forward	Fus intron 7	GGAGAAACTGGATGGATGCAC
mFUS-Exon 8/9-R1	Reverse	Fus exons 8 and 9	CCTGTTCAGAATCATGACGAGA
mSRSF7 F1	Forward	Srsf7 exon 4	CGACGAAGAAGAAGCAGGTTTC
mSRSF7 R1	Reverse	Srsf7 exon 5	TCTGGCCTCTTATGCTGATCAC
mXIST F1	Forward	Xist	CAGAGTAGCGAGGACTTGAAGAG
mXIST R1	Reverse	Xist	GCTGGTTCGTCTATCTTGTGGG
mFUS-Exon 9	Reverse	Fus exon 9 (for fractionation)	GCCTTGCACGAAGATGGTATT

Supplementary Table 6: Primer sequences used for RT-PCR in human

Name	Orientation	Target	Sequence (5′-3′)
hFUS-Exon 6-F1	Forward	FUS exon 6	TCCTCCATGAGTAGTGGTGGT
hFUS-Intron 6-R4	Reverse	FUS intron 6	GTTCAGGCTCCCAAGTTCTC
hFUS-Intron 7-F3	Forward	FUS intron 7	TTCTCTCGGGTGAGAGAACC
hFUS-Exon 8/9-R2	Reverse	FUS exons 8 and 9	GTCTGAATTATCCTGTTCGGAGTC

Supplementary Table 7: primers used for RT-qPCR in human cells

Name	Orientation	Localisation	Sequence (5′-3′)
FUS Ex1F	Forward	Exon 1	AGCGGTGTTGGAACTTCG
FUS Ex3R	Reverse	Exon 3	GACTGCTCTGCTGGGAATAG
FUS Ex6F	Forward	Exon 6	CAGCAGTGGTGGCTATGAAC
FUS Ex9R	Reverse	Exon 9	TGCACAAAGATGGTGTTGTTG
b-actin	Forward	Exon 5	TCCATCATGAAGTGTGACGT
b-actin	Reverse	Exon 6	TACTCCTGCTTGCTGATCCAC
hnRNPL NMD	Forward	NMD exon	GGTCGCAGTGTATGTTTGATG
hnRNPL NMD	Reverse	Exon 3	GGCGTTTGTTGGGGTTGCT

Supplementary Table 8: Characteristics of other RNA-seq datasets used

Dataset	Citation/accession	Library Prep	Read length
Human iPSCs differentiated into motor neurons +- VCP mutations	Luisier et al, 2018 GSE98290	polyA+, Truseq stranded	75bp single end
Human motor neurons +- SOD1 mutations	Kiskinis et al, 2014 GSE54409	polyA+, Truseq v2 stranded	100bp paired end
Human FTD post-mortem brain	Swarup et al, 2019 GSE90696	Ribodepletion, Truseq stranded	50bp paired end
Human ALS post-mortem brain	Prudencio et al, 2015 GSE67196	Ribodepletion, Truseq stranded	100bp paired end

Supplementary Figures

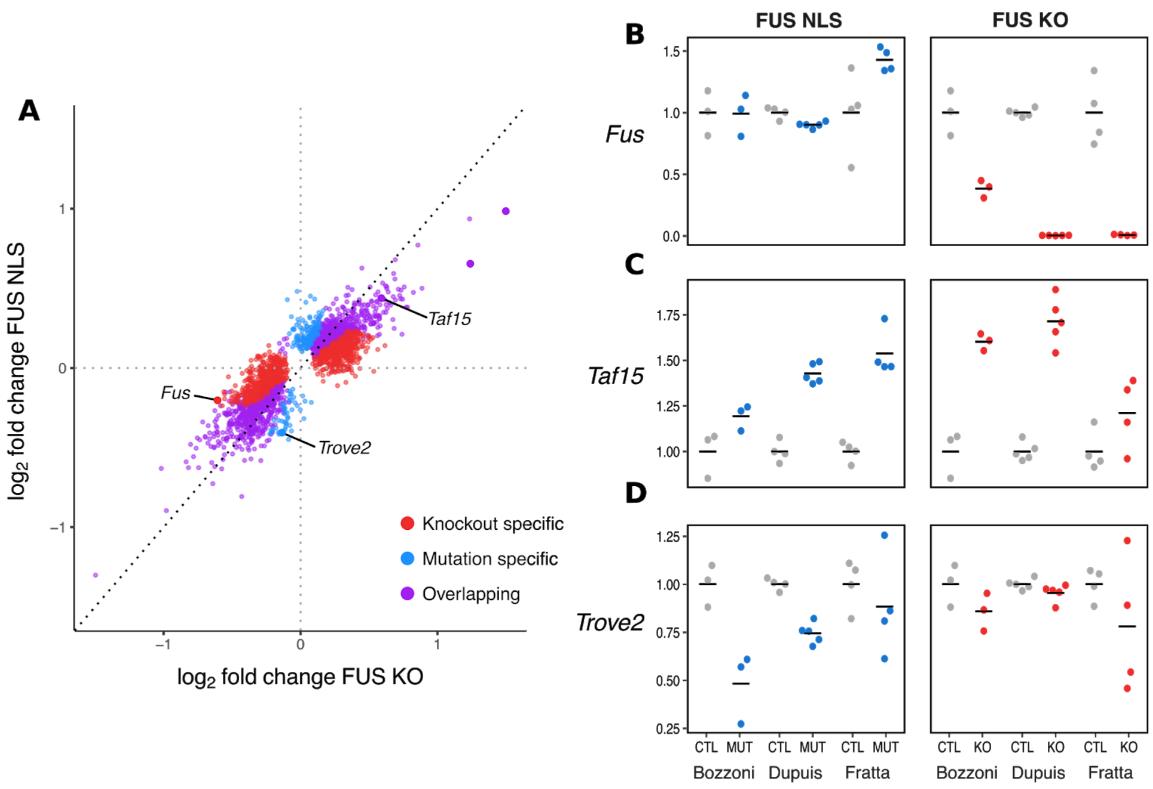

Supplementary Figure 1: Overlapping gene expression models show concordant effects of FUS KO and FUS NLS mutations

(**A**) Log_2 fold change of FUS KO joint model plotted against log_2 fold change in FUS NLS joint model for all three categories of genes. (**B-D**) Expression plots for *Fus, Taf15,* and *Trove2* in each sample of each dataset. Library size-normalised expression in FUS NLS (MUT) and FUS KO samples is normalised to that of each set of wildtype littermates (CTL).

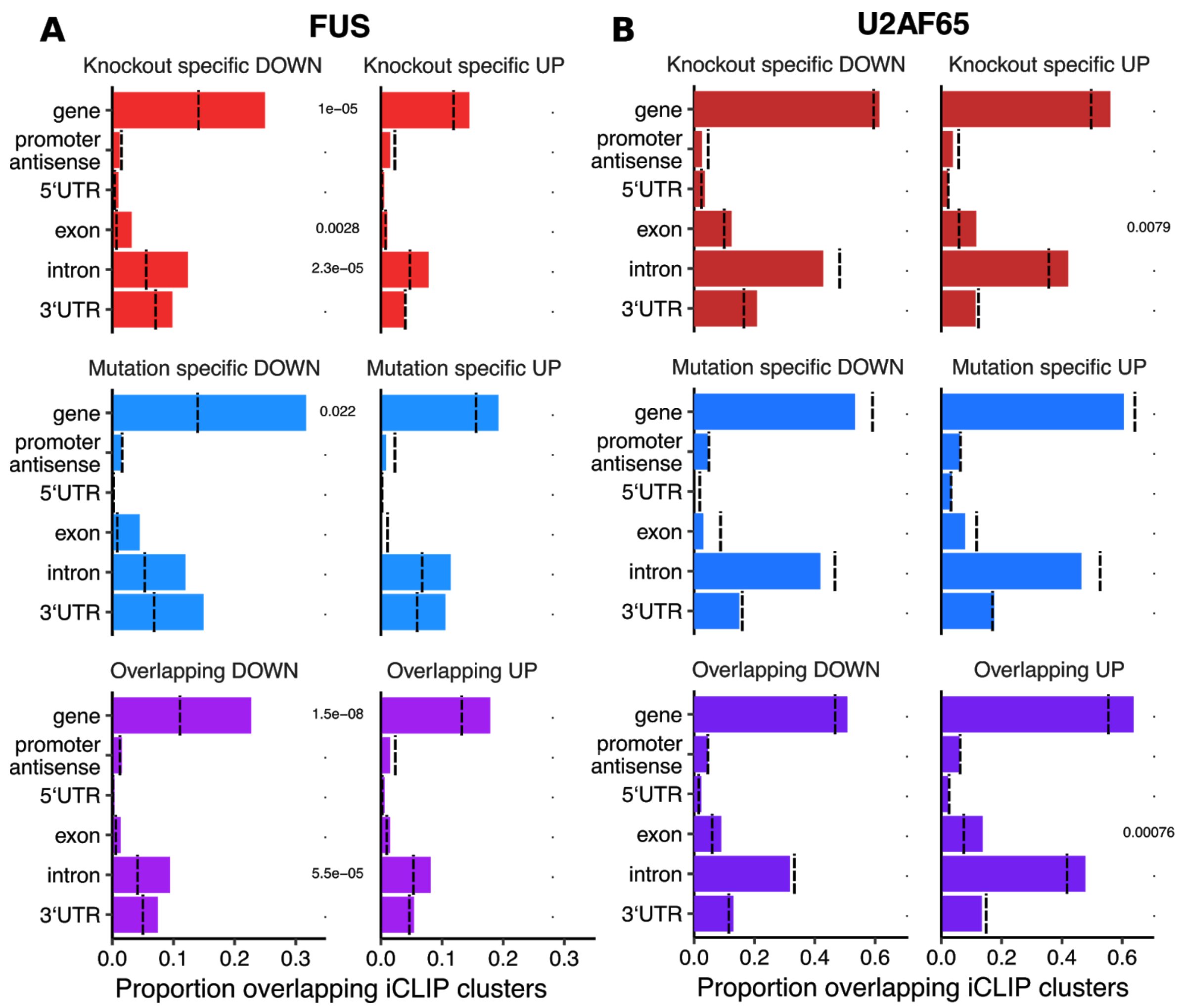

Supplementary Figure 2: Downregulated genes are enriched in FUS iCLIP clusters

(**A**) Proportion of genes that overlap with a FUS iCLIP cluster. Genes divided by direction of change and group (Knockout-specific, Mutation-specific and Overlapping). Proportions in null sets depicted with black dotted lines. (**B**) As above, but for U2AF65 iCLIP. All P-values corrected for multiple testing with Bonferroni method. Any P-value > 0.05 not shown.

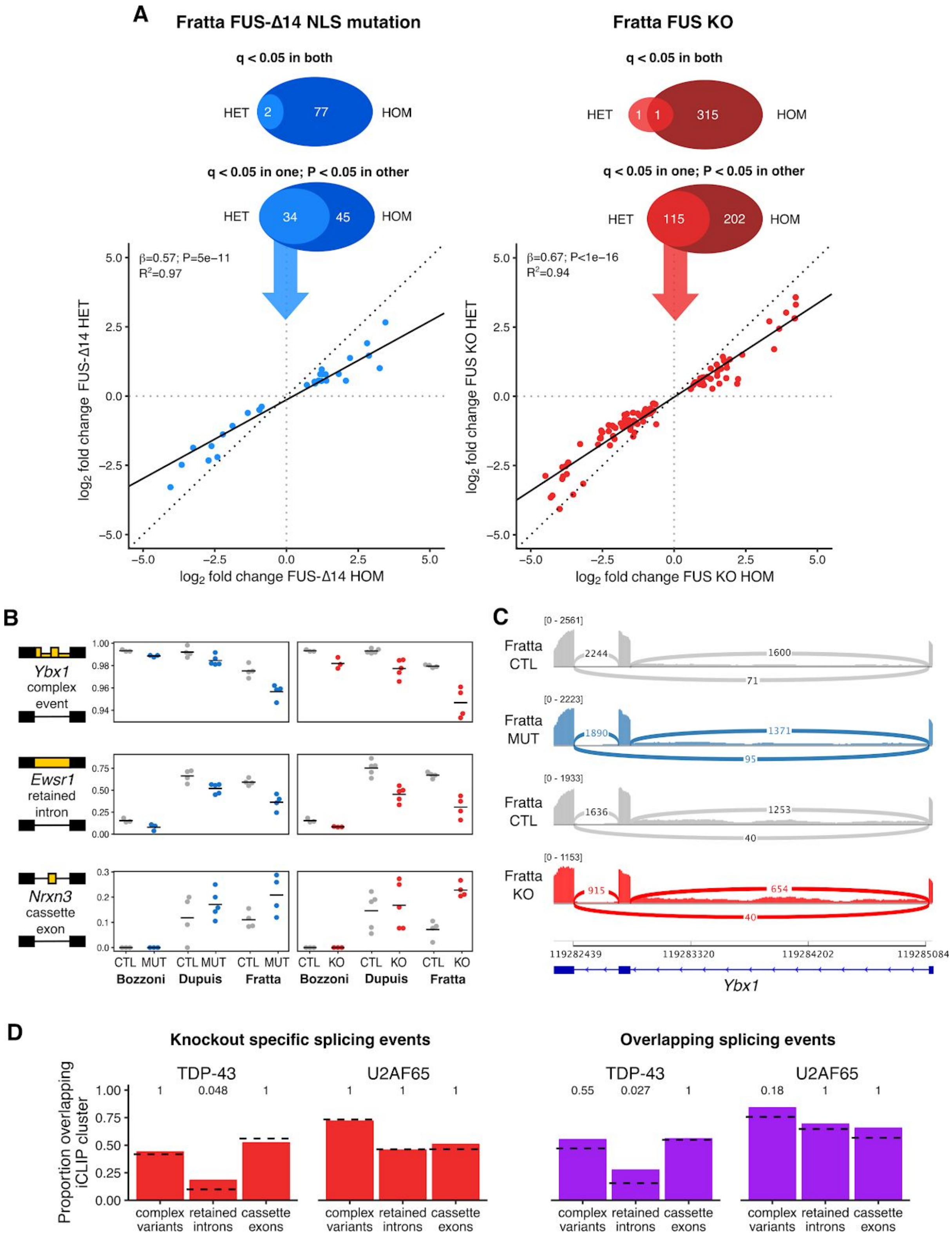

Supplementary Figure 3: Accompaniment to splicing analyses

(**A**) Comparing splicing events found in Fratta FUS-Δ14 and FUS KO homozygous samples with those found in heterozygous samples. (**B**) Percentage spliced in (PSI) for three splicing events in the six comparisons. (**C**) Representative RNA-seq traces for a complex event in *Ybx1* in the Fratta FUS-Δ14 NLS mutation and FUS KO samples with their respective

controls. (**D**) Proportions of knockout-specific and overlapping splicing events in each category that contain TDP-43 or U2AF65 iCLIP peaks. P-values corrected for multiple testing with Bonferroni method.

A mouse Fus intron 6

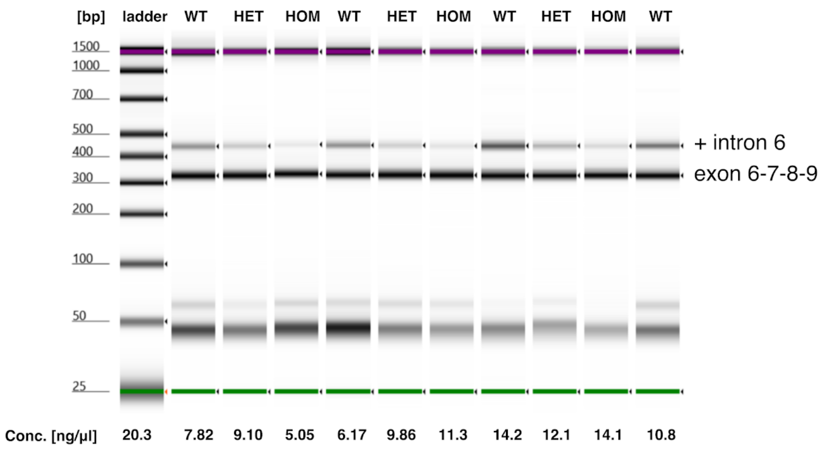

B Mouse Fus intron 7

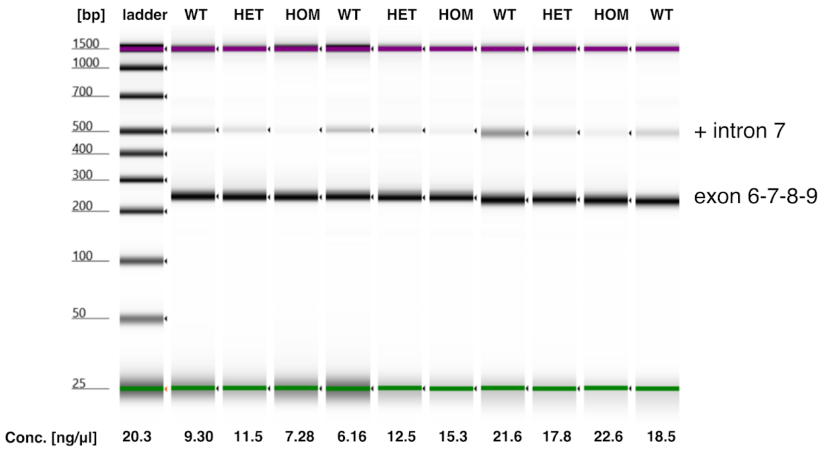

Supplementary Figure 4: RT-PCR of *FUS* intron retention in mouse

(A) TapeStation traces from RT-PCR with two primers targeting *FUS* mRNA between exons 6 and 9 and a third primer targeting *FUS* intron 6. Samples with wildtype *FUS* (WT), heterozygous (HET) or homozygous FUS-Δ14 (HOM). RNA concentrations are taken from the TapeStation. Bands used in quantification are annotated. **(B)** As before for *FUS* intron 7.

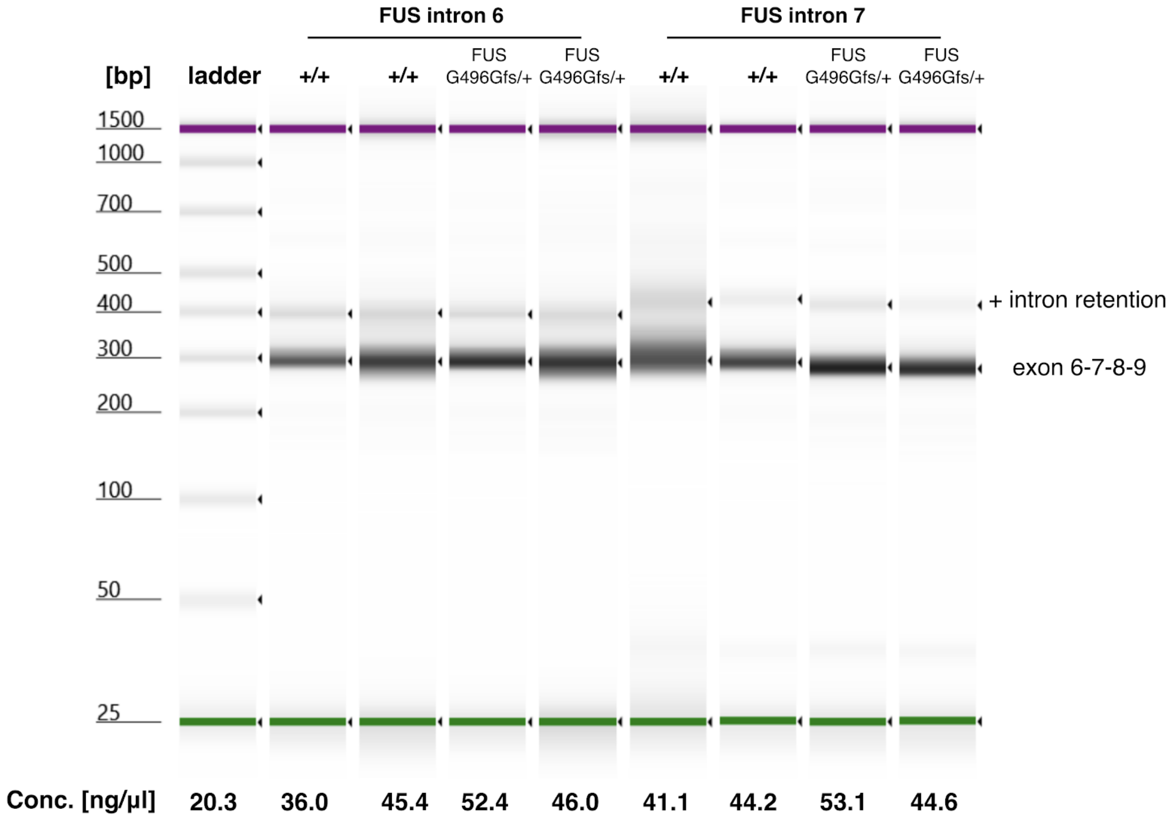

Supplementary Figure 5: RT-PCR of *FUS* intron retention in human samples

TapeStation traces from RT-PCR with two primers targeting *FUS* mRNA between exons 6 and 9 and a third primer targeting *FUS* intron 6 (left 4 lanes) or intron 7 (right 4 lanes). Samples taken from a patient with wildtype *FUS* (+/+) and a patient heterozygous for the *FUS* G496Gfs mutation (G496Gfs/+). Replicates are technical, derived from separate RNA extractions. RNA concentrations are taken from the TapeStation itself. Bands used in quantification are annotated.

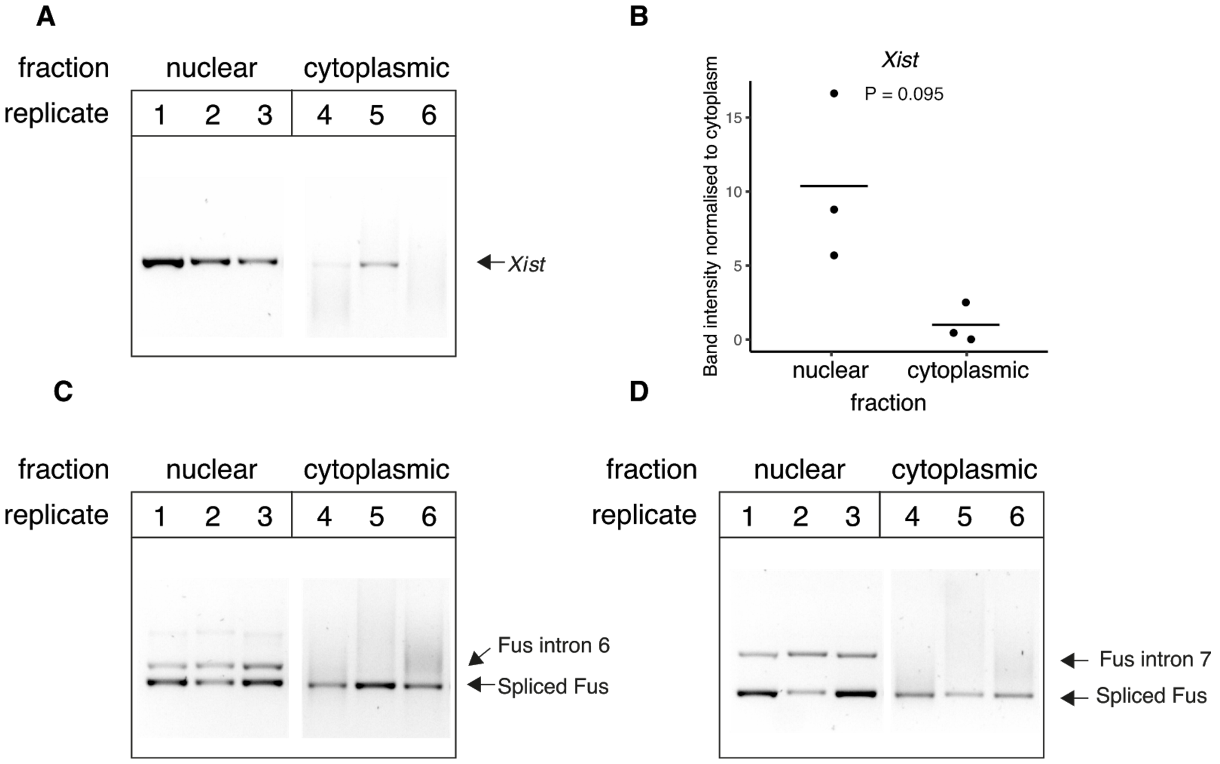

Supplementary Figure 6: Nuclear and cytoplasmic fractionation

(A) Agarose gel containing the RT-PCR of Xist in nuclear fraction and cytoplasmic fraction. (B) Quantification of *Xist* products, with band intensities normalised to mean of the cytoplasmic fraction. (C) Agarose gel from the three-primer RT-PCR of *FUS* intron 6 in the two fractions. (D) As before, for the *FUS* intron 7. Lanes in each panel are derived from the same gel - lanes not relevant to this work have been omitted.

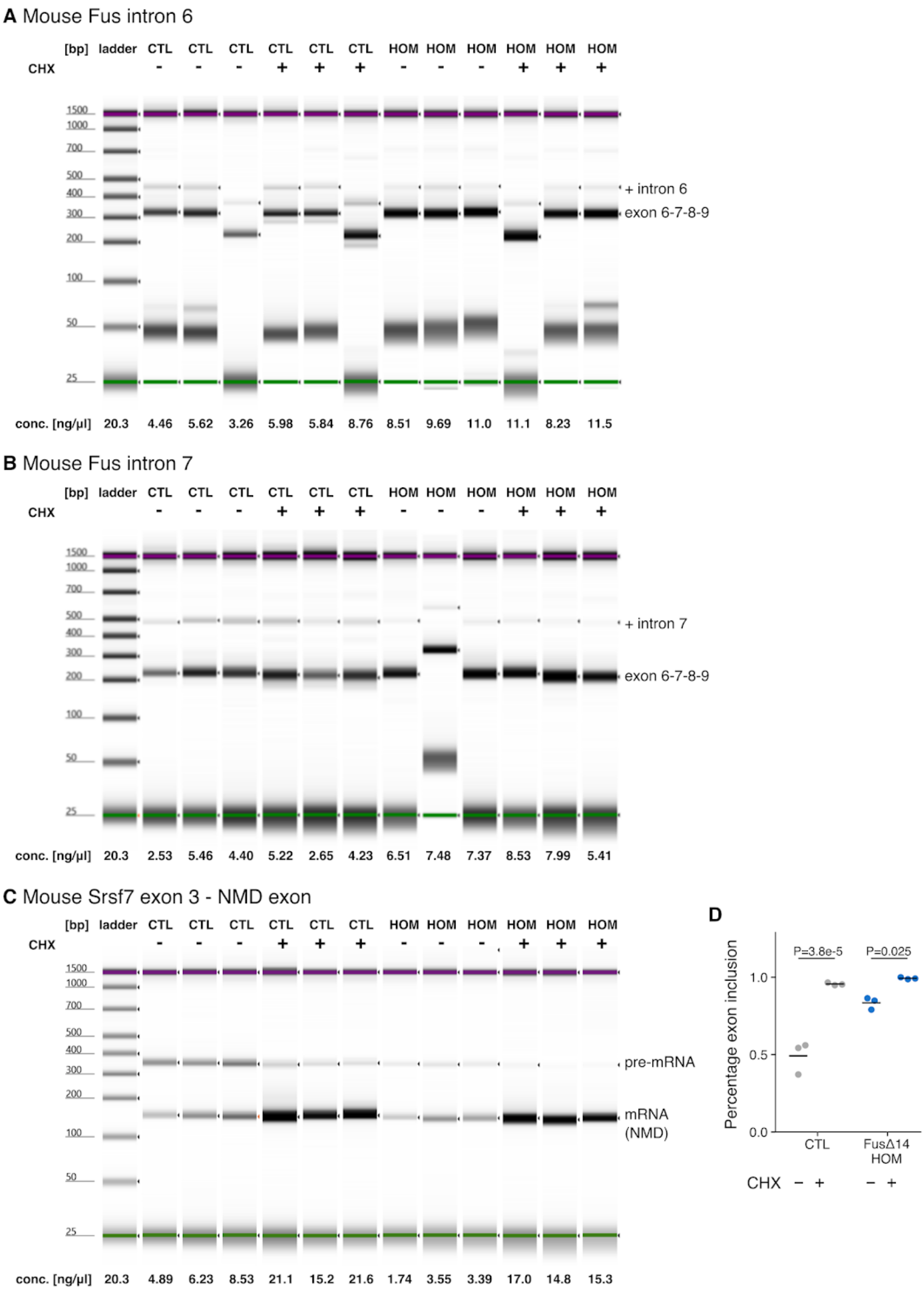
A Mouse Fus intron 6
[bp] ladder CTL CTL CTL CTL CTL CTL HOM HOM HOM HOM HOM HOM
CHX - - - + + + - - - + + +
1500 1000 700 500 400 300 200 100 50 25
+ intron 6
exon 6-7-8-9
conc. [ng/µl] 20.3 4.46 5.62 3.26 5.98 5.84 8.76 8.51 9.69 11.0 11.1 8.23 11.5
B Mouse Fus intron 7
[bp] ladder CTL CTL CTL CTL CTL CTL HOM HOM HOM HOM HOM HOM
CHX - - - + + + - - - + + +
1500 1000 700 500 400 300 200 100 50 25
+ intron 7
exon 6-7-8-9
conc. [ng/µl] 20.3 2.53 5.46 4.40 5.22 2.65 4.23 6.51 7.48 7.37 8.53 7.99 5.41
C Mouse Srsf7 exon 3 - NMD exon
[bp] ladder CTL CTL CTL CTL CTL CTL HOM HOM HOM HOM HOM HOM
CHX - - - + + + - - - + + +
1500 1000 700 500 400 300 200 100 50 25
pre-mRNA
mRNA
(NMD)
conc. [ng/µl] 20.3 4.89 6.23 8.53 21.1 15.2 21.6 1.74 3.55 3.39 17.0 14.8 15.3
D
Percentage exon inclusion
P=3.8e-5
P=0.025
1.0
0.5
0.0
CTL
FusΔ14
HOM
CHX − + − +

Supplementary Figure 7: Cycloheximide inhibition experiments

(A) TapeStation traces from RT-PCR with primers targeting *Fus* intron 6. Samples with wildtype *FUS* (CTL) or homozygous *FUS*-Δ14 (HOM) with or without treatment with Cycloheximide (CHX). RNA concentrations are taken from the TapeStation itself. Bands used in quantification are annotated. (B) As before, but for Fus intron 6. (C) As before but amplifying spliced and unspliced RNA between *Srsf7* exon 3 and its known NMD exon. (D) Quantification of *Srsf7* mRNA against total. ANOVA treatment P = 1.1e-5; genotype P = 3.7e-4; interaction P = 1.4e-3

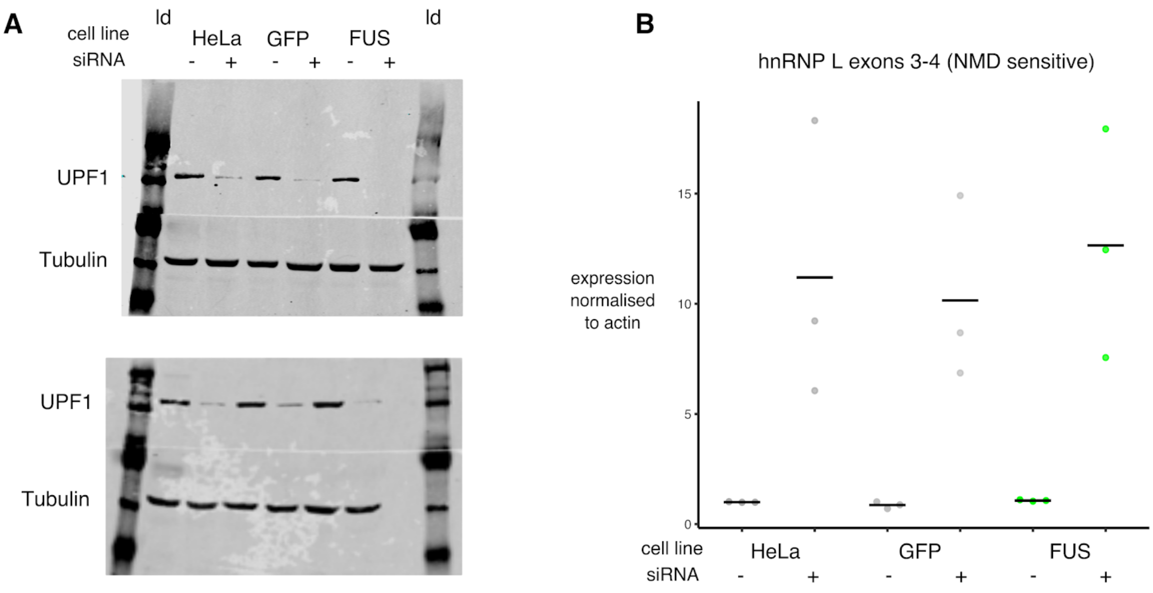

Supplementary Figure 8: FUS overexpression qPCR and UPF1 knockdown

(**A**) Two independent western blotting experiments demonstrate efficient knockdown of UPF1 protein by siRNA. Tubulin used as a loading control. ld: protein ladder. (**B**) Quantification of expression of hnRNP L NMD-sensitive transcript by RT-qPCR demonstrates UPF1 knockdown is sufficient to inhibit NMD. HeLa - control HeLa cell line; GFP - HeLa cells expressing GFP construct; FUS - HeLa cells expressing codon-optimised FUS transcript. + denotes UPF1 siRNA, - denotes a scrambled siRNA.

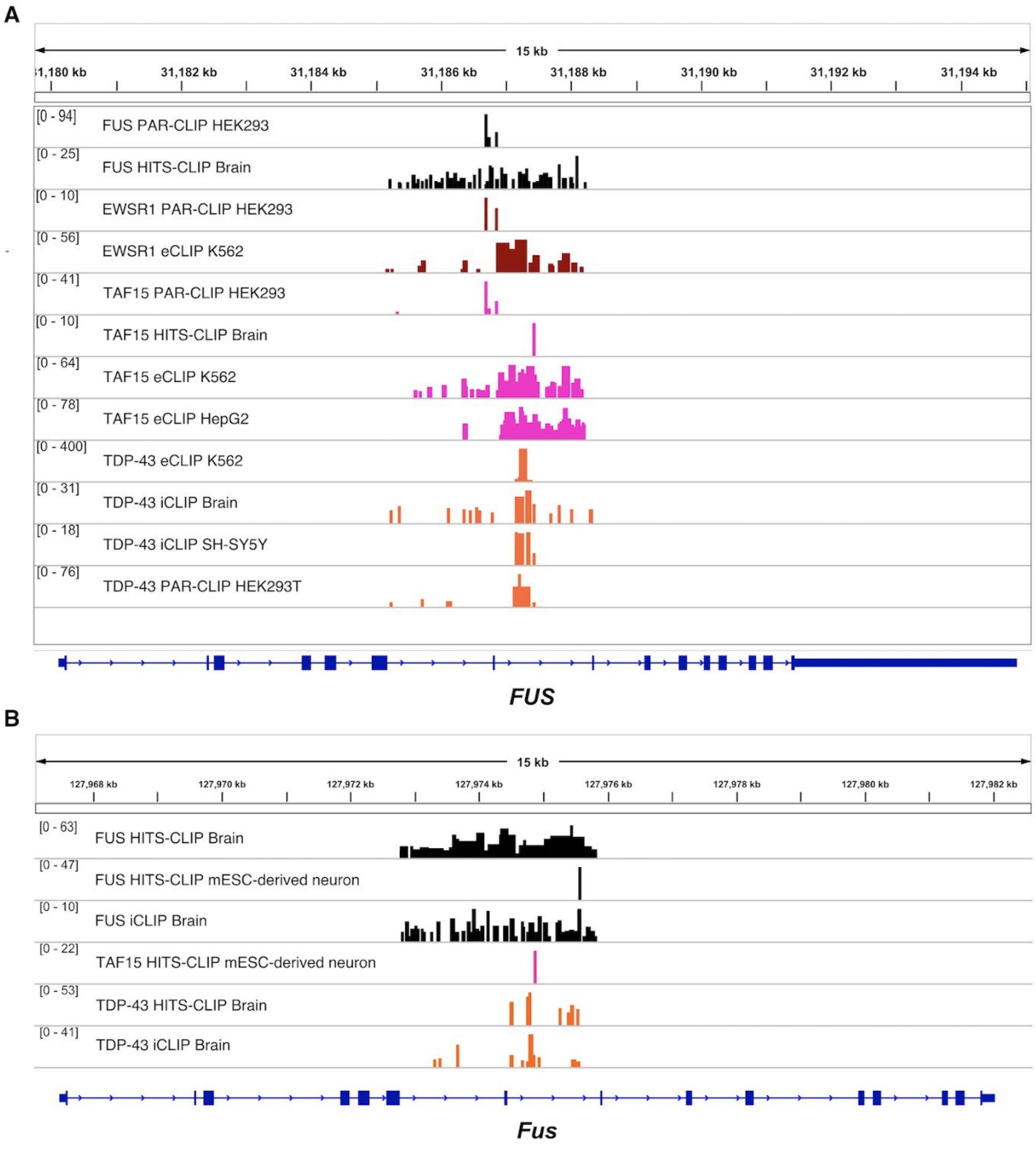

Supplementary Figure 9: FUS, EWSR1, TAF15 and TDP-43 bind FUS introns 6 and 7 in human and mouse

(**A**) IGV traces across the human *FUS* gene of all CLIP data from the POSTAR/CLIPdb database of the 4 RBPs. Scale indicates the maximum number of reads. (**B**) IGV traces across the mouse *Fus* gene. No mouse EWSR1 CLIP was present in the POSTAR/CLIPdb database.

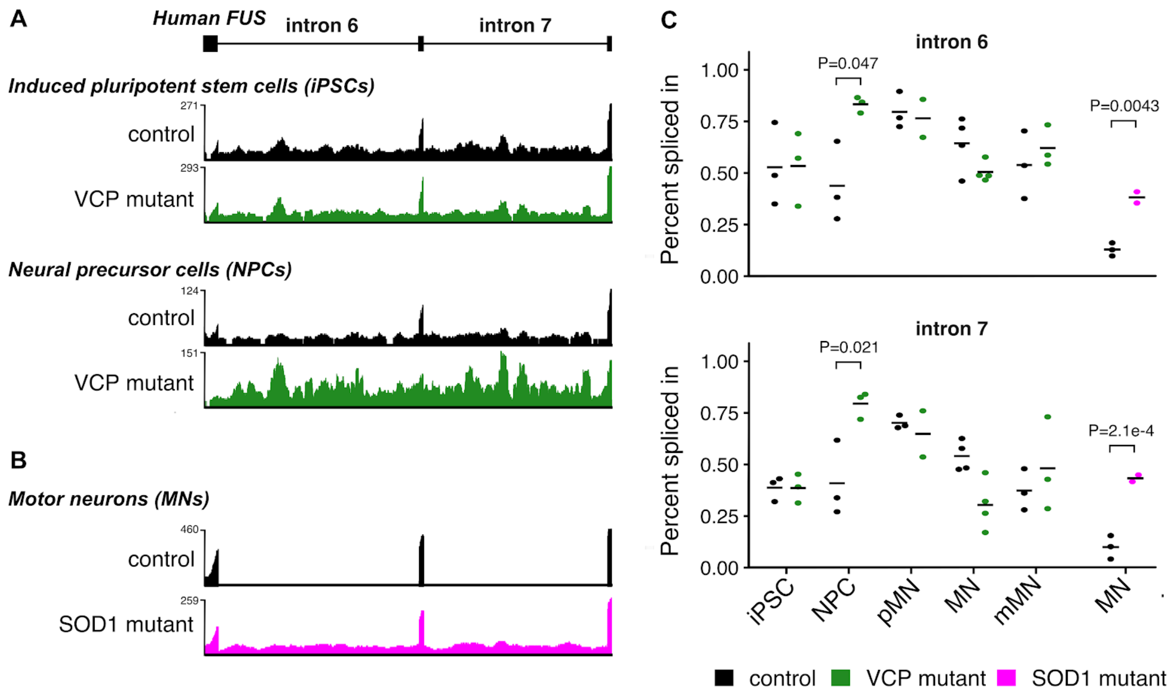

Supplementary Figure 10: FUS intron retention is dysregulated in different ALS models

(**A**) RNA-seq traces from human induced pluripotent stem cells and neural precursor cells with and without mutations in VCP show a selective increase in retention of introns 6 and 7. Data from Luisier et al, 2017 (**B**) RNA-seq traces from motor neurons (MN) with and without the A4V mutation in SOD1. (**C**) Percent spliced in quantification of all samples across neural differentiation from induced pluripotent stem cell, neural precursor cell (NPC), precursor motor neuron (pMN), immature motor neuron (MN) and mature motor neuron (mMN). ANOVA on PSI ~ cell type + genotype intron 6 P=0.019 intron 7 P=0.00052. Individual -values from Tukey post-hoc test. For the MN samples with and without SOD1 mutations, P-values were taken directly from the splicing analysis.

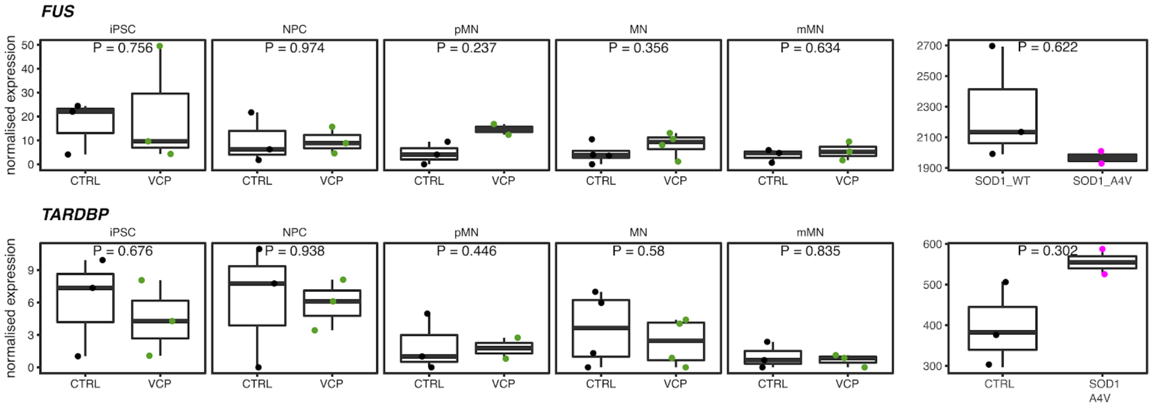

Supplementary Figure 11: FUS and TARDBP expression changes in VCP and SOD1 mutations

Plots show normalised gene expression values for FUS (top panel) and TARDBP, the TDP-43 transcript (bottom panel), in Luisier et al VCP dataset (left) and the Kiskinis et al SOD1 human motoneuron dataset (right-most panels). Neither gene is significantly differentially expressed in any comparison. P-values taken from DESeq2.

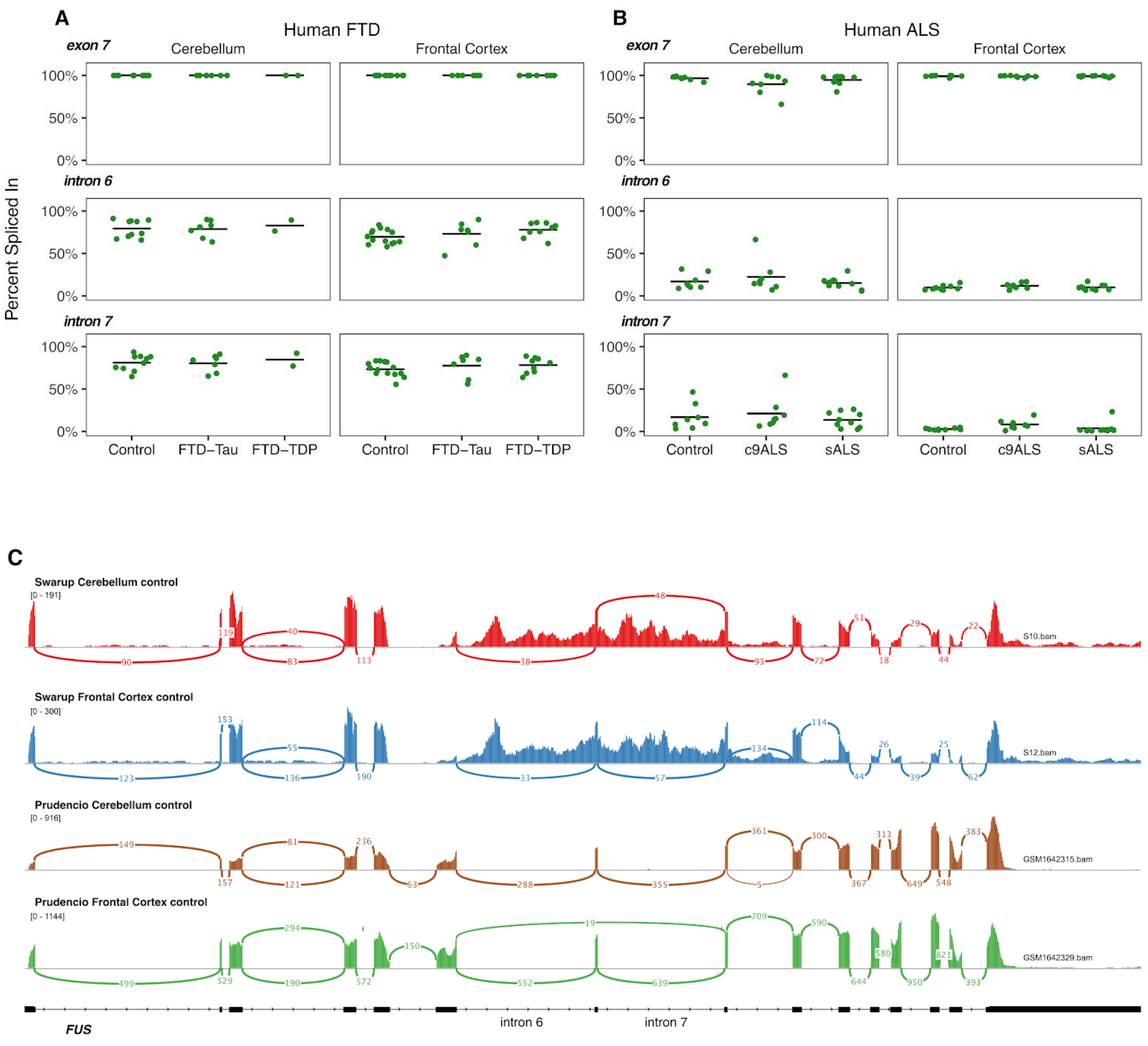

Supplementary Figure 12: FUS intron retention in human ALS and FTD post-mortem brain

(A) Percentage spliced in values for human FUS exon 7, intron 6 and intron 7 in human cerebellum (left panels) and frontal cortex (right panels). No difference in FUS splicing observed between FTD-Tau nor FTD-TDP cases compared to controls. Data from Swarup et al, 2018. **(B)** As above but comparing ALS cases with C9orf72 expansions (c9ALS) and sporadic ALS cases (sALS) with controls again shows no difference in FUS splicing. Data from Prudencio et al, 2015. **(C)** Representative control samples from cerebellum and frontal cortex from both datasets visualised as sashimi plots using the IGV browser confirms differences in FUS intron retention between the two datasets and the negligible skipping of exon 7. Numbers in square brackets refer to the maximum read coverage depth for each sample. Numbers between the junctions refer to the number of junction reads for that sample at that junction. Original sample ID on the right of each trace.
